# Regulation of CD8+ T cells by lipid metabolism in cancer progression

**DOI:** 10.1038/s41423-024-01224-z

**Published:** 2024-10-14

**Authors:** Yong Tang, Ziqing Chen, Qianying Zuo, Yibin Kang

**Affiliations:** 1 Department of Molecular Biology, Princeton University, Princeton, NJ 08544 USA; 2 Ludwig Institute for Cancer Research Princeton Branch, Princeton, NJ 08544 USA; 3 Cancer Metabolism and Growth Program, Rutgers Cancer Institute of New Jersey, New Brunswick, NJ 08903 USA

**Keywords:** CD8+T cells, Lipid metabolism, Mitochondria, Oxidative phosphorylation, Immunotherapy, Tumour immunology, Cancer metabolism

## Abstract

Dysregulation of lipid metabolism is a key characteristic of the tumor microenvironment, where tumor cells utilize lipids for proliferation, survival, metastasis, and evasion of immune surveillance. Lipid metabolism has become a critical regulator of CD8+ T-cell-mediated antitumor immunity, with excess lipids in the tumor microenvironment impeding CD8+ T-cell activities. Considering the limited efficacy of immunotherapy in many solid tumors, targeting lipid metabolism to enhance CD8+ T-cell effector functions could significantly improve immunotherapy outcomes. In this review, we examine recent findings on how lipid metabolic processes, including lipid uptake, synthesis, and oxidation, regulate CD8+ T cells within tumors. We also assessed the impact of different lipids on CD8+ T-cell-mediated antitumor immunity, with a particular focus on how lipid metabolism affects mitochondrial function in tumor-infiltrating CD8+ T cells. Furthermore, as cancer is a systemic disease, we examined systemic factors linking lipid metabolism to CD8+ T-cell effector function. Finally, we summarize current therapeutic approaches that target lipid metabolism to increase antitumor immunity and enhance immunotherapy. Understanding the molecular and functional interplay between lipid metabolism and CD8+ T cells offers promising therapeutic opportunities for cancer treatment.

## Introduction

Cancer is the second leading cause of death worldwide and is expected to become the leading cause of mortality globally soon after 2030 [1]. Decades of research have led to the realization that cancer is a complex and systemic disease driven by a combination of tumor-intrinsic, microenvironmental and systemic factors [2]. Tumors emerge and progress within a dynamic microenvironment composed of diverse cell types, the extracellular matrix, growth factors, cytokines, chemokines, and metabolites. Alterations in this microenvironment significantly impact tumor growth and metastasis. Additionally, systemic changes in the body, such as aging and obesity, influence tumor progression.

CD8+ T cells are crucial for suppressing tumor growth and metastasis, but their functions are often compromised in the TME. This dysfunction is caused primarily by two factors: immunosuppressive signals from tumor cells or other suppressive cells, along with the limited availability of oxygen and nutrients, which affect the metabolic features of CD8+ T cells and their antitumor functions. CD8+ T cells are central to immunotherapies such as immune checkpoint blockade (ICB) and adoptive cell transfer (ACT), which enhance the immune response against tumors. Despite the success of these therapies in leukemia and certain solid tumors, their efficacy remains limited in most solid tumors, particularly those that are metastatic [3].

Metabolic reprogramming is a hallmark of cancer [4]. In the tumor microenvironment, tumor cells preferentially convert glucose to lactate even when oxygen is sufficient, a phenomenon known as the Warburg effect. This metabolic shift facilitates rapid ATP generation and supplies intermediates for biosynthetic pathways, thereby supporting cell growth, proliferation, and metastasis. While glucose is the primary metabolic substrate for most cells in tumors, other substrates, including amino acids, glycolytic metabolites, and lipids, are also utilized. Various lipid species, such as cholesterol, fatty acids, and triacylglycerols, are abundant in different tumor tissues. Lipids serve as energy sources, membrane components, and signaling molecules and play complex roles in tumor progression, including both tumor-promoting and tumor-suppressive effects.

In this review, we aim to highlight the lipid metabolic features of CD8+ T cells in tumor progression. By advancing our understanding of lipid metabolism regulation in CD8+ T cells within the context of cancer, we hope to inform the development of metabolic therapies that could increase the efficacy of immunotherapies for cancer patients.

## Lipid homeostasis regulation

**Fig. 1:**
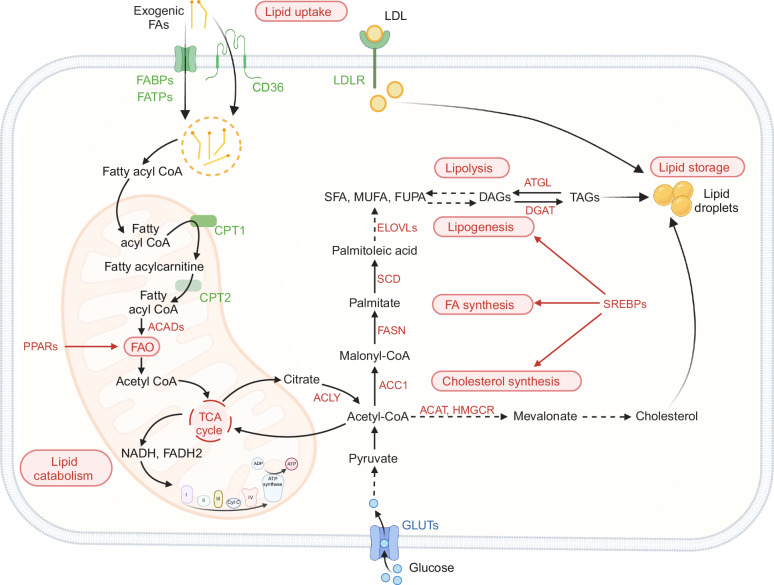
Lipid metabolism in cells. This figure illustrates the pathways of lipid metabolism in cells. Fatty acids (FAs) enter the cell through lipid translocases such as CD36, FABPs, and FATPs or via passive diffusion. Low-density lipoprotein (LDL) enters the cell through low-density lipoprotein receptors (LDLRs). Inside the cell, FAs are esterified to acyl-CoAs by ACSL enzymes for metabolism. Acyl-CoAs destined for catabolism are transported into mitochondria through CPTs to undergo fatty acid oxidation (FAO). Acetyl-CoA produced from FAO enters the TCA cycle, with electrons from NADH and FADH2 used in oxidative phosphorylation (OXPHOS) for ATP generation and oxygen respiration. Citrate from the TCA cycle can exit the mitochondria and be converted into acetyl-CoA by ACLY, initiating de novo fatty acid synthesis. Acetyl-CoA can be used by HMGCR for cholesterol synthesis or by FASN, along with malonyl-CoA, to produce palmitate, which is then activated to palmitoyl-CoA. Activated palmitate can be desaturated by SCDs to create monounsaturated and polyunsaturated fatty acids (MUFAs and PUFAs) and elongated by ELOVLs. Fatty acyl-CoAs combine with glycerol to form monoacylglycerols (MAGs), diacylglycerols (DAGs), and finally triacylglycerols (TAGs) via DGAT. TAGs are stored in lipid droplets, and lipases release FAs from TAGs, DAGs, and MAGs through hydrolysis. The figure also highlights the main transcriptional programs for lipid catabolism (PPARs) and anabolism (SREBPs)

Lipid metabolism is a complex physiological process crucial for nutrient balance, hormonal regulation, and homeostasis. It includes the synthesis of various structural and functional lipids, such as phospholipids, glycolipids, sphingolipids, cholesterol, and prostaglandins, with some showing tissue-specific distribution patterns [5]. This process also involves lipid breakdown to meet the body’s metabolic demands, such as energy production (Fig. 1). Lipid metabolism maintains a dynamic equilibrium, with some lipids being oxidized to satisfy metabolic needs while others are synthesized and stored [6]. Lipids are vital for ATP production and are involved in synthesizing vitamins, hormones, bile salts, eicosanoids, and cellular membranes. They also play a significant role in regulating cellular signaling [7]. For example, cholesterol and phospholipids are integral components of cellular membranes and are crucial for maintaining their fluidity, permeability, and structural integrity [8, 9]. Lipid anabolism and catabolism are compartmentalized within the cell (Fig. 1). Anabolism, the synthesis of lipids, primarily occurs in the cytosol and endoplasmic reticulum (ER). In contrast, catabolism, the breakdown of lipids, mainly takes place in the mitochondria. This compartmentalization ensures that lipid metabolism is tightly regulated and efficiently meets the body’s metabolic demands [10].

### Lipogenesis and storage

Lipogenesis, the process of fatty acid and subsequent triglyceride synthesis, occurs primarily in the liver and adipose tissue [11, 12]. This process is highly sensitive to dietary changes. A carbohydrate-rich diet stimulates lipogenesis in the liver and adipose tissue, leading to elevated postprandial plasma triglyceride levels [13]. Glucose, a key substrate for lipogenesis, is converted glycolytically to acetyl-CoA, promoting fatty acid synthesis [14]. Additionally, glucose induces the expression of lipogenic genes, such as sterol regulatory element-binding proteins (SREBPs) and peroxisome proliferator-activated receptors (PPARs). The body stores fat in the form of adipose triacylglycerols (TAGs), which are used for heat production, energy, and insulation [15]. Fasting reduces lipogenesis in adipocytes and, combined with increased lipolysis, results in a net loss of triglycerides from fat cells [16].

De novo fatty acid synthesis (FAS) occurs in the cytosol (Fig. 1) and is initiated by the activation of SREBP1 [17, 18]. During this process, mitochondrial citrate acts as a precursor to generate palmitate (16:0), which can then be desaturated to palmitoleic acid (16:1) by stearoyl-CoA desaturase (SCD) and elongated by members of the elongation of long-chain fatty acids (ELOVL) family [19]. FAS is driven by several enzymes, including acetyl-CoA citrate lyase (ACLY), acetyl-CoA carboxylase 1 (ACC1), and fatty acid synthase (FASN). ACC1 plays a crucial role by carboxylating carbohydrate-derived acetyl-CoA in the cytosol to produce malonyl-CoA, the essential precursor for FAS. Through the generation of malonyl-CoA, ACC1 initiates the first committed step in fatty acid synthesis, thereby supporting the proliferation and function of CD8+ T cells under both homeostatic and inflammatory conditions [20]. Stimulation of the T-cell receptor (TCR) activates the PI3K-Akt and mechanistic target of rapamycin (mTOR) signaling pathways to induce fatty acid and mevalonate synthesis [21–23].

Most mammalian cells acquire cholesterol from the bloodstream via low-density lipoprotein (LDL) receptors (LDLRs). LDLs are derived from very low-density lipoproteins (VLDLs) produced by the liver, following the gradual release of their triglyceride content [24]. In addition to being synthesized via dietary uptake, cholesterol can be synthesized in most mammalian cells from acetyl-CoA. Briefly, three molecules of acetyl-CoA condense to form 3-hydroxy-3-methyl-glutaryl-CoA (HMG-CoA), which is then converted to mevalonate by the rate-limiting enzyme HMG-CoA reductase (HMGCR) [8, 24, 25].

### Lipid uptake and catabolism

Lipolysis is a metabolic process in which TAGs are broken down through hydrolysis into glycerol and free fatty acids (FFAs) [15]. Fatty acid oxidation (FAO), also known as β-oxidation, is a metabolic process in which long-chain fatty acids are broken down in the mitochondria to produce acetyl-CoA, which is then utilized for energy production in the tricarboxylic acid (TCA) cycle and electron transport chain [26]. Fatty acid translocase/cluster of differentiation 36 (CD36) is a crucial membrane glycoprotein responsible for importing fatty acids into cells [27]. The family of fatty acid transport proteins (FATPs, also collectively known as SLC27) and plasma membrane fatty acid-binding proteins (FABPs) also regulate fatty acid import, transport, and metabolism [27]. Elevated levels of FFAs have been associated with breast cancer, promoting tumor growth and metastasis [28–31].

The mitochondrial membrane is impermeable to acyl-CoAs. Fatty acids are transported into the mitochondrial matrix through the carnitine cycle, which involves the coordinated action of carnitine acyltransferase I (CPT1), the rate-limiting enzyme of β-oxidation located in the outer mitochondrial membrane, and carnitine acyltransferase II (CPT2) and carnitine-acylcarnitine translocase in the inner mitochondrial membrane [32]. Malonyl-CoA regulates fatty acid β-oxidation by inhibiting CPT1 activity, thereby preventing the import of fatty acids into the mitochondrial matrix for β-oxidation [33, 34].

Inside the mitochondria, acyl-CoAs undergo β-oxidation, a cyclic process involving four steps: dehydrogenation to trans-2-enoyl-CoA, hydration to (S)-3-hydroxyacyl-CoA, dehydrogenation to 3-ketoacyl-CoA, and cleavage to acetyl-CoA and a shortened acyl-CoA chain (Fig. 1). This process generates acetyl-CoA for the TCA cycle and ketogenesis and produces flavin adenine dinucleotide (FADH2) and reduces nicotinamide adenine dinucleotide (NADH) for the electron transport chain. β-Oxidation enzymes are specific to different chain lengths of fatty acids to ensure complete degradation [35]. The acyl-CoA dehydrogenase family comprises at least 11 enzymes predominantly involved in FAO. Each FAO cycle is initiated by an acyl-coenzyme A dehydrogenase (ACAD) enzyme. In humans, key ACAD enzymes include very long-chain, medium-chain, and short-chain acyl-CoA dehydrogenases (VLCAD, MCAD, and SCAD, respectively), each with specific roles in FAO [36]. FAO is regulated by both transcriptional and posttranscriptional mechanisms. PPARα, PPARβ/δ, and PPARγ are ligand-activated nuclear receptors that form heterodimers with the retinoid X receptor and regulate fatty acid metabolism transcriptionally. PPARs are activated by fatty acids and play tissue-specific roles [37]. PPARα governs hepatic FAO gene expression [37, 38], while PPARα and PPARβ/δ together regulate FAO enzyme expression in skeletal muscle and the heart, and PPARγ functions primarily in adipocytes [39, 40].

## Metabolic features of CD8+ T cells

CD8+ T cells, also known as cytotoxic T cells, are critical components of the adaptive immune system. They play a pivotal role in the body’s defense against intracellular pathogens and the destruction of tumor cells. Naïve CD8+ T cells begin the activation process upon encountering an antigen-presenting cell that displays a specific viral or tumor antigen via major histocompatibility complex class I (MHC I) molecules. Numerous studies have indicated that nutrients and metabolic pathways are crucial regulators of CD8+ T-cell activation. As key components of cell membranes and sources of energy and signaling molecules, lipids play a significant role in CD8+ T-cell activation and function through lipid metabolism. In the tumor microenvironment, elevated lipid levels can drive dysfunction in effector CD8+ T cells, leading to their exhaustion [41, 42].

**Fig. 2:**
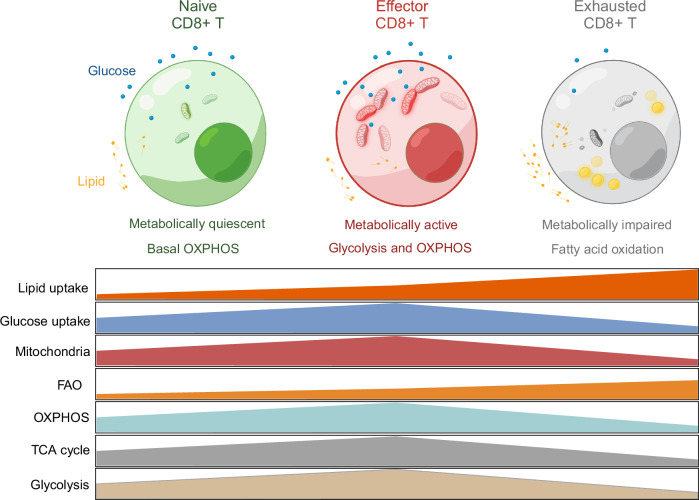
Metabolic features of different CD8+ T cells. This figure depicts the distinct metabolic characteristics of CD8+ T cells in different states. Naïve CD8+ T cells are metabolically quiescent and take up minimal amounts of glucose and lipids to maintain their slow metabolic activities. These cells primarily derive their energy from the TCA cycle, with OXPHOS playing a significant role. Upon activation, effector CD8+ T cells become highly metabolically active and increase their mitochondrial activities. To meet the increased energy demands for proliferation and effector functions, these cells increase their uptake of glucose and lipids. Effector CD8+ T cells rely primarily on glycolysis and OXPHOS for energy production, sustaining their rapid growth and cytotoxic activities. In the tumor microenvironment, CD8+ T cells often become exhausted, resulting in impaired metabolism. Exhausted CD8+ T cells have reduced activities in both the TCA cycle and OXPHOS, relying predominantly on FAO to meet their energy needs

Different lineages of CD8+ T cells exhibit distinct metabolic features (Fig. 2). Naïve CD8+ T cells remain in a quiescent state and take up minimal amounts of glucose and lipids from the surrounding environment. With low energy demands for survival and proliferation, they maintain slow metabolic activity. In naïve CD8+ T cells, ATP production primarily results from the oxidation of pyruvate in the TCA cycle, oxidative phosphorylation (OXPHOS), and fatty acid oxidation (FAO) [43]. Their mitochondria are usually relatively small in size [44]. Upon activation, naïve CD8+ T cells differentiate into effector CD8+ T cells. To meet the increased demands for activation and proliferation, effector CD8+ T cells predominantly utilize glycolysis. This metabolic shift allows them to quickly generate the large amounts of energy and biomass required for their rapid expansion and function [45]. They also increase OXPHOS to produce additional ATP. Interestingly, while effector CD8+ T cells take up more lipids, they also need de novo lipogenesis to achieve optimal T-cell function under both homeostatic and inflammatory conditions [20]. In contrast to effector CD8+ T cells, memory CD8+ T cells rely less on the uptake of extracellular fatty acids. Instead, they utilize extracellular glucose to support FAO and OXPHOS for their development, which are critical for their development [46]. In the tumor microenvironment, effector CD8+ T cells can be exhausted through various regulatory pathways. Owing to the limited availability of glucose and other nutrients in the tumor microenvironment, exhausted CD8+ T cells reduce their glycolytic activity. Mitochondrial function is often impaired, leading to decreased OXPHOS. Consequently, exhausted CD8+ T cells increasingly rely on FAO for energy generation to maintain their effector function [47]. Moreover, some studies have shown that most endogenous tumor-infiltrating T cells (TILs) exhibit repressed mitochondrial biogenesis and restricted capacity to utilize FAO [48–50], which can further dampen T-cell effector function. Interestingly, a subset of exhausted T cells known as precursors of exhausted T (Tpex) cells, which retain memory-like characteristics and progenitor potential [51, 52], exhibit high mitochondrial activity and spare respiratory capacity [53]. These findings highlight the complex interplay between energy metabolism and T-cell differentiation. However, how lipid metabolism regulates the metabolic and functional heterogeneity of exhausted T cells remains unclear. Overall, lipid metabolism and homeostasis are critical for maintaining CD8+ T-cell function during tumor progression.

## CD8+ T cells and lipid metabolism

### Lipid uptake in CD8+ T cells

The tumor microenvironment is enriched with various types of lipids, which CD8+ T cells take up through different lipid transporters. CD36, a scavenger receptor, is well known for transporting fatty acids and oxidized lipids, such as oxidized low-density lipoproteins (OxLDLs) and phosphocholine-containing phospholipids (OxPLs) [54]. Studies in mouse models and human samples have shown that CD36 expression increases in tumor-infiltrating CD8+ T cells, dampening their function [42]. High CD36 expression in tumor tissues is correlated with poor survival in various cancer types. Elevated cholesterol levels in the tumor microenvironment upregulate CD36 expression in tumor-infiltrating CD8+ T cells, inducing lipid peroxidation and ferroptosis, thereby impairing their effector function. Genetic deletion of CD36 or pharmacological inhibition of ferroptosis in CD8+ T cells can restore their cytotoxicity and synergize with anti-PD-1 immunotherapy [41].

FABPs are a family of transport proteins that facilitate the transport and utilization of fatty acids within cells [55]. In hepatocellular carcinoma (HCC), CD137 expression identifies a subset of exhausted T cells with superior effector function and proliferation capacity. These CD137+ exhausted T cells expressed elevated levels of FABP5 and displayed increased mitochondrial OXPHOS. The presence of FABP5-high CD8+ T cells in tumors is associated with better overall and recurrence-free survival. Inhibiting FABP5 expression and mitochondrial FAO promotes apoptosis and reduces the proliferation of CD137-enriched exhausted CD8+ T cells [56]. Interestingly, tissue-resident memory (TRM) CD8+ T cells express different isoforms of FABP depending on their location [57, 58]. Skin CD8+ TRM cells upregulate FABP4/5 to take up fatty acids after infection [59], whereas liver CD8+ TRM cells express high levels of FABP1 [57]. Without FABP1, T cells are unable to accumulate in the liver, although this does not affect their distribution in the spleen [57]. These findings suggest that fatty acid metabolism might play a key role in enabling T cells to adapt to tissue-specific environments, supporting their survival and persistence in tissues with abundant fatty acids, such as the liver or skin. Moreover, CD8+ T cells infiltrating skin tumors also take up and metabolize fatty acids, resulting in increased expression of FABP4/5 [47]. Thus, targeting the FABP family could represent a promising therapeutic strategy for enhancing antitumor immunity and improving cancer treatment outcomes.

FATPs are a family of transmembrane proteins that increase long-chain fatty acid (LCFA) uptake and are expressed in all fatty acid-utilizing tissues [60]. In multiple myeloma, decreased mitochondrial mass and markedly elevated LCFA uptake reduce bone marrow CD8+ T-cell function. High FATP1 expression in bone marrow CD8+ T cells is responsible for increased LCFA uptake, and FATP1 blockade rescues CD8+ T-cell function [61]. LDLR mainly mediates the uptake of LDL cholesterol. LDLR was significantly increased in activated CD8+ T cells but not in CD4+ T cells. LDLR knockout in CD8+ T cells significantly decreases cell proliferation and cytokine production [62]. LDLR not only plays a role in T-cell priming and clonal expansion but also interacts with the TCR to regulate TCR cycling and signaling. This interaction enhances the cytotoxicity of CD8+ T cells in tumors. Proprotein convertase subtilisin/kexin type 9 (PCSK9) regulates cholesterol metabolism by binding to LDLR, preventing the recycling of LDLR and TCR to the plasma membrane and thus inhibiting CD8+ T-cell effector function. Genetic deletion or pharmacological inhibition of PCSK9 in tumor cells enhances the antitumor activity of CD8+ T cells, indicating that the PCSK9/LDLR axis is a potential therapeutic target for cancer immunotherapy [63].

### Lipid synthesis in CD8+ T cells

CD8+ T cells can also synthesize lipids de novo, primarily via acetyl-CoA. Enzymes involved in lipid synthesis are critical for maintaining CD8+ T-cell function in nutrient-deficient environments [64]. IL-12-stimulated CD8+ T cells maintain high levels of IFNγ in an ACLY-dependent manner. ACLY increases the level of intracellular acetyl-CoA, improving metabolic and functional fitness by increasing histone acetylation and lipid synthesis. Inhibiting ACLY severely impairs IFNγ production and CD8+ T-cell viability under nutrient-restricted conditions [65]. Tumor-infiltrating CD8+ T cells exhibit elevated lipid storage due to increased ACC activity. Restricting ACC activity rewires T-cell metabolism, enhances survival and polyfunctionality, and suppresses tumor growth [50]. FASN, the key enzyme in fatty acid synthesis, plays an essential role in the function and survival of memory CD8+ T cells. These cells rely on fatty acid synthesis for their long-term persistence, and inhibition of FASN results in decreased survival of memory CD8+ T cells, while effector CD8+ T cells are largely unaffected [46]. However, the specific role of FASN in tumor-infiltrating CD8+ T cells remains poorly understood and warrants further investigation to determine its impact on their antitumor function. SREBPs activate genes encoding enzymes required for cholesterol and unsaturated fatty acid synthesis [66]. SREBPs coordinate TCR signaling with lipid-anabolic programs necessary for rapid membrane biosynthesis and cellular growth. Inhibiting SREBP signaling reduces CD8+ T-cell proliferative capacity in vitro and clonal expansion during viral infection [21]. Tumor regulatory T cells (Tregs) promote M2-like tumor-associated macrophages by suppressing IFNγ production by CD8+ T cells, which blocks SREBP1-dependent fatty acid metabolism in macrophages. Inhibiting SREBP1 augments the efficacy of anti-PD-1 immunotherapy [67]. However, the exact role of SREBP signaling in CD8+ T cells under tumor conditions remains largely unknown and requires further investigation.

### Lipid oxidation in CD8+ T cells

In response to nutrient limitations in the tumor microenvironment, CD8+ T cells, despite their ability to take up more glucose than tumor cells [68], can also utilize lipids as alternative energy sources through fatty acid oxidation. Increasing lipid oxidation can enhance the efficacy of adoptively transferred CD8+ effector T cells or PD-1 blockade therapies [69]. PPARs are ligand-activated transcription factors belonging to the nuclear hormone receptor superfamily that are involved in the regulation of energy homeostasis. PPARα, the major isoform expressed in lymphocytes, activates fatty acid oxidation pathways and is the target of hypolipidemic fibrate drugs [70]. Interestingly, while both skin CD8+ TRM cells and CD8+ T cells infiltrating skin tumors share similarities in terms of lipid uptake, they differ in their FAO regulation ability because of the expression of different PPARs. Skin CD8+ TRMs express PPARγ [59], whereas CD8+ T cells infiltrating skin tumors express PPARα [47]. In response to conditions such as hypoglycemia and hypoxia, tumor-infiltrating CD8+ T cells increase PPARα signaling and fatty acid catabolism to maintain energy production and effector functions. PPARα agonists, such as fenofibrate and bezafibrate, improve CD8+ T-cell functions and synergize with PD-1 blockade to inhibit tumor growth [47, 71].

Signal transducer and activator of transcription 3 (STAT3) is a transcription factor with broad effects on controlling tumor growth and the immune response [72]. STAT3 signaling is essential for promoting the development of intratumor-terminally exhausted CD8+ T cells by enhancing their effector functions and survival, leading to better tumor control [73]. Both PD-1 ligation and enrichment of leptin in mammary tissues can activate STAT3 signaling in tumor-infiltrating CD8+ T cells, driving increased FAO, which is critical for obesity-associated breast tumor progression [74]. IL-9/STAT3-mediated FAO contributes to IL-9-producing CD8+ T-cell (Tc9) longevity and enhanced antitumor activity. IL-9 activates STAT3, which increases FAO and mitochondrial activity, protecting CD8+ T cells from tumors or reactive oxygen species (ROS)-induced ferroptosis [75].

CPT regulates the β-oxidation of long-chain fatty acids and catalyzes the transfer of the acyl group from coenzyme A to carnitine to form palmitoylcarnitine. The CPT1 family has three isoforms that are distributed in different tissues [76], and CPT1A is the major isoform in lymphocytes. Ex vivo treatment with the CPT1 inhibitor etomoxir significantly reduces FAO but increases the expression of glycolytic genes in CD8+ T cells. Additionally, another CPT1 inhibitor, perhexiline, has been shown to inhibit breast tumor growth in obese mice. More tumor-infiltrating CD8+ T cells produce IFNγ and Granzyme B in perhexiline-treated groups [74]. However, etomoxir has off-target effects on T-cell metabolism, which may limit its application. Using genetic mouse models, researchers have demonstrated that CPT1A mediates long-chain fatty acid oxidation but is not required for effector and memory T-cell responses [77]. Further characterization in genetic mouse and tumor models is needed to understand the role of CPT1A-mediated FAO in the CD8+ T-cell-regulated antitumor response.

In summary, lipid metabolism plays an essential role in regulating the function and efficacy of CD8+ T cells within the TME. Understanding the mechanisms of lipid uptake, synthesis, and oxidation in these cells provides valuable insights into potential therapeutic strategies to enhance antitumor immunity. Targeting specific lipid metabolic pathways offers promising avenues for improving cancer immunotherapy outcomes.

## Specific lipids in the reulation of CD8+ T Cells

Lipids are structurally diverse and functionally varied. In the context of cancer, understanding the specific function of individual lipids or lipid types is crucial for designing therapeutic strategies aimed at enhancing CD8+ T-cell effector function in tumors. This section explores the impact of different lipid types on CD8+ T-cell function within the tumor microenvironment, highlighting their potential as therapeutic targets to enhance antitumor immunity.

### Fatty acids

Fatty acids are categorized by their chain length: short-chain fatty acids (SCFAs), medium-chain fatty acids (MCFAs), long-chain fatty acids (LCFAs), and very-long-chain fatty acids (VLCFAs). Additionally, they are classified as saturated fatty acids (SFAs) or unsaturated fatty acids (UFAs), which include both monounsaturated (MUFAs) and polyunsaturated (PUFAs) fatty acids. The composition and specific characteristics of these fatty acid classes, along with their degree of saturation, play critical roles in regulating the proliferation, survival, and antitumor functions of tumor-infiltrating CD8+ T cells.

The gut microbiota profoundly influences the cytotoxicity of tumor-infiltrating CD8+ T cells and impacts the clinical efficacy of immune checkpoint therapies [78]. Butyrate, an SCFA secreted by the gut microbiota, enhances mTOR function and inhibits class I histone deacetylase activity in CD8+ T cells or chimeric antigen receptor (CAR) T cells, leading to elevated effector cytokine production and improved antitumor immunity [79]. Additionally, microbial butyrate directly enhances the CD8+ T-cell-mediated antitumor immune response through the inhibition of DNA binding 2 (ID2)-dependent IL-12 signaling [80]. Moreover, butyrate enhances the memory potential of activated CD8+ T cells by promoting metabolic adaptations, such as uncoupling the TCA cycle from glycolytic inputs [81].

In pancreatic tumors, CD8+ T cells progressively accumulate LCFAs, impairing mitochondrial function and reducing fatty acid catabolism. In vitro treatment with palmitic acid (a common LCFA) dampens CD8+ T-cell proliferation and effector cytokine production [82]. The desaturase SCD1 converts stearic acid and palmitic acid into UFAs, such as oleic acid and palmitoleic acid, respectively. SCD1 deficiency in CD8+ T cells drives their differentiation into effector T cells, although this can be partially reversed by supplementation with oleic acid [83]. Oleic acid (a MUFA) and linoleic acid (a PUFA) are both common in dietary oils. However, only a linoleic acid-rich high-fat diet (HFD) has been shown to promote breast tumor growth, despite both oleic and linoleic acids inducing similar obesity in mouse models, highlighting the distinct effects of MUFAs and PUFAs on tumor progression. Epidermal fatty acid binding protein (E-FABP) expression in naïve CD8+ T cells facilitates linoleic acid transport via mitochondria and its incorporation into cardiolipin, which induces lipid peroxidation and mitochondrial ROS production, leading to apoptosis and reduced TNFα production [84]. Interestingly, linoleic acid has also been shown to redirect tumor-infiltrating CD8+ T cells from an exhausted phenotype toward a memory-like state, enhancing their effector functions. These findings suggest that linoleic acid treatment could serve as a potential booster for adoptive T-cell transfer therapies [85].

Arachidonic acid (a PUFA), derived from linoleic acid, regulates calcium signaling in CD4+ T cells and promotes synovial inflammation in rheumatoid arthritis patients [86]. In the tumor microenvironment, arachidonic acid, in combination with IFNγ produced by CD8+ T cells, induces tumor ferroptosis in an ACSL4-dependent manner [87]. However, arachidonic acid can also induce ferroptosis in tumor-infiltrating CD8+ T cells via CD36 expression [41]. In addition to these effects, fatty acids also support the fitness and functionality of tumor-resident CD8+ T cells by maintaining SCML4 (scm polycomb group protein like 4) expression, increasing the therapeutic effect of anti-PD-1 treatment [88].

Prostaglandins are a group of PUFAs derived from arachidonic acid, with prostaglandin E2 (PGE2) being a key lipid compound that exerts broad immune regulatory functions by binding to EP2/4 receptors. PGE2 levels are elevated in human tumor tissues and impair IL-2 sensing in human CD8+ T cells by downregulating the IL-2 receptor gamma chain (IL-2Rg_c_), leading to oxidative stress and ferroptosis in tumor-reactive lymphocytes. Pharmacological inhibition of PGE2 signaling during TIL expansion for adoptive cell transfer enhances the proliferation of tumor-reactive TILs and improves tumor control [89]. Tumor-derived PGE2 also restricts the proliferation and effector differentiation of TCF1+ stem-like CD8+ T cells by suppressing the IL-2 signaling pathway. Genetic ablation of the PGE2 receptors EP2/EP4 in CD8+ T cells restores their effector functions, eliminating tumor growth in mouse models [90]. In addition to prostaglandins, other PUFAs, including eicosapentaenoic acid (EPA), docosahexaenoic acid (DHA), and docosapentaenoic acid (DPA), can also modulate T-cell activity in the tumor microenvironment [91]. For example, dietary SFAs can promote tumor progression, whereas dietary PUFAs generally do not impact tumor growth [92]. Further research is needed to fully understand the mechanisms by which SFAs and UFAs influence CD8+ T-cell-mediated antitumor immunity.

Collectively, these insights emphasize the nuanced role of different fatty acids in fine-tuning CD8+ T-cell activity and shaping immune responses in cancer, suggesting potential therapeutic avenues for targeting lipid metabolism to increase immunotherapy efficacy.

### Cholesterol and derivatives

Cholesterol plays a critical role in maintaining the structural integrity of cell membranes and the plasma compartment. It directly binds to the transmembrane domain of the TCR-beta chain, facilitating TCR clustering on the T-cell surface, which enhances TCR signaling avidity toward foreign antigens and amplifies TCR signaling [93]. Tumor cells can modulate cholesterol metabolism in CD8+ T cells through PCSK9, which inhibits LDLR-mediated recycling of the TCR, thereby impairing TCR signaling [63]. PCSK9 also suppresses CD8+ T effector function by downregulating MHC I expression on tumor cells, independent of its role in cholesterol regulation [94]. Acetyl-CoA acetyltransferase 1 (ACAT1), an enzyme involved in cholesterol esterification, is crucial for regulating cholesterol levels [95]. Inhibiting ACAT1 increases plasma membrane cholesterol levels in CD8+ T cells, leading to increased TCR clustering and signaling, which boosts CD8+ T-cell effector function and proliferation. Therefore, ACAT1 inhibition has been shown to enhance antitumor immunity and improve the efficacy of anti-PD-1 therapy [96]. Cholesterol also plays a role in suppressing the differentiation and function of Tc9 cells by inhibiting IL-9 expression through the activation of liver X receptors (LXRs). This activation leads to LXR sumoylation, reducing the binding of p65 to the IL-9 promoter and thereby dampening the Tc9 cell-mediated antitumor immune response [97]. The cholesterol derivative 27-hydroxycholsestrerol, produced primarily by the enzyme CYP27A1, promotes tumor progression by increasing the number of polymorphonuclear neutrophils and γδ-T cells while decreasing the number of cytotoxic CD8+ T cells, which facilitates breast cancer metastasis. Inhibition of CYP27A1 significantly suppresses metastasis [98].

Cholesterol levels vary significantly across different cell types within the tumor microenvironment. While tumor cells and immunosuppressive myeloid cells tend to accumulate cholesterol [99, 100], tumor-infiltrating CD8+ T cells often face cholesterol deficiency, potentially due to competition for cholesterol or altered metabolic pathways [96]. This imbalance creates a dual challenge: excess cholesterol in CD8+ T cells can induce exhaustion through ER stress and the upregulation of immune checkpoints such as PD-1 [101]. Conversely, oxysterols mediate reciprocal alterations in the LXR and SREBP2 pathways, leading to cholesterol deficiency in CD8+ T cells. This deficiency contributes to CD8+ T-cell dysfunction and induces autophagy-mediated apoptosis, further impairing their antitumor activity [102]. Thus, within the TME, cholesterol dysregulation can simultaneously lead to CD8+ T-cell exhaustion via accumulation and impaired T-cell survival via deficiency, exacerbating immune suppression and promoting tumor progression. This complex interplay highlights the need for targeted metabolic interventions to restore cholesterol homeostasis and improve T-cell functionality in cancer immunotherapy.

### Other lipids

Ceramide, a sphingolipid family member, is a major component of cell membrane lipid bilayers and serves as a signaling molecule [103]. Acid sphingomyelinase (Asm) hydrolyzes sphingomyelin to ceramide, which is further metabolized to sphingosine by acid ceramidase (Ac). T-cell-specific knockout of Asm, which reduces ceramide levels in T cells, results in enhanced melanoma tumor progression and an impaired T-cell immune response. Conversely, T-cell-specific Ac-deficient mice presented the opposite results. Ceramide colocalizes with the TCR and CD3 on the membrane of stimulated T cells, increasing the phosphorylation of TCR signaling molecules [104].

Lysophosphatidic acid (LPA), a bioactive lysophospholipid, contributes to tumorigenesis. LPA regulates diverse functions through binding to G protein-coupled receptors (GPCRs), including LPA receptor 1-6 (LPAR1-6). LPA inhibits CD8+ T-cell function via the LPAR5 receptor, regulating CD8+ T-cell respiration, proton leakage, and reactive oxygen species, reprogramming T-cell metabolism to modulate antitumor immunity [105]. Additionally, LPA levels predict the response to immunotherapy and can serve as a lipid-regulated immune checkpoint [106].

In conclusion, specific lipids play critical roles in regulating CD8+ T-cell function, suggesting potential targets for enhancing antitumor immunity and improving immunotherapy outcomes. Understanding the complex interplay between different lipids and CD8+ T cells is essential for developing effective cancer treatments.

## Lipid regulation on mitochondria in CD8+ T cells

**Fig. 3:**
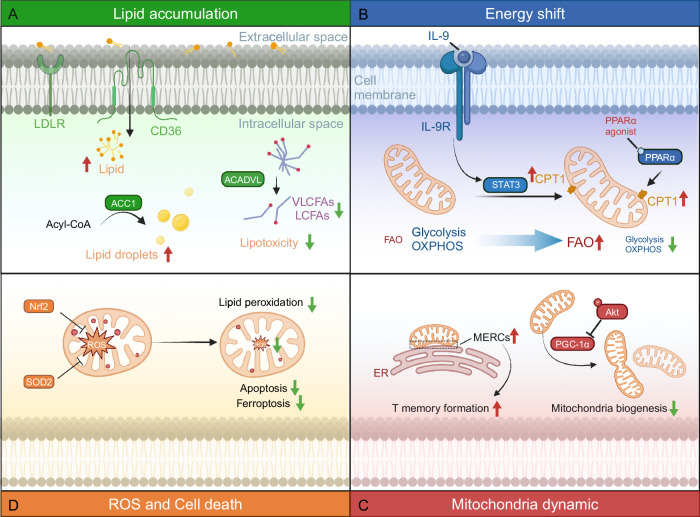
Effects of lipid metabolism on mitochondria in tumor-infiltrating CD8+ T cells. This figure shows how lipid metabolism impacts mitochondrial function in tumor-infiltrating CD8+ T cells through various mechanisms. **A** Lipid accumulation. Tumor-infiltrating CD8+ T cells upregulate CD36 and LDLR to increase lipid uptake. Increased ACC activity leads to the formation of lipid droplets, whereas increased ACADVL expression reduces the levels of LCFAs and VLCFAs, decreasing lipotoxicity. **B** Shifts in energy metabolism. The IL-9/STAT3/CPT1 axis shifts energy metabolism in tumor-infiltrating CD8+ T cells from glycolysis/OXPHOS to FAO, increasing cytotoxicity. PPARα agonists activate PPARα to increase FAO. **C** Disruptions in mitochondrial dynamics. Akt inhibits PGC-1α, suppressing mitochondrial biogenesis. Increased MERCs in tumor-infiltrating CD8+ T cells promote memory T-cell formation. **D** Increased ROS production and cell death. Nrf2 or SOD2 inhibits ROS production, reducing lipid peroxidation, apoptosis, and ferroptosis in tumor-infiltrating CD8+ T cells

Mitochondria, the metabolic hubs of cells, play crucial roles in regulating lipid metabolism. A proteomics study of 116 melanoma patients undergoing immunotherapy indicated that a positive response to treatment was associated with enriched mitochondrial lipid metabolism in tumor cells. High mitochondrial metabolism in tumor cells leads to increased antigen presentation and interferon signaling, increasing T-cell cytotoxicity against tumor cells [107]. Mitochondrial metabolism supports T-cell anabolism by providing key metabolites for macromolecule synthesis and generating energy for T-cell function [108]. However, the lipid-enriched tumor microenvironment can impair mitochondrial function in infiltrated CD8+ T cells, leading to reduced OXPHOS, increased ROS production, and even cell death. This section discusses how lipids in the tumor microenvironment modulate mitochondrial function in CD8+ T cells (Fig. 3).

### Lipid accumulation

Excessive lipid accumulation is frequently observed in tumor-infiltrating CD8+ T cells, contributing to their dysfunction and exhaustion. To adapt to the lipid-enriched tumor microenvironment, CD8+ T cells upregulate lipid transporters to obtain lipids as an energy source under nutrient stress. For example, the upregulation of CD36 and LDLR increases the uptake of extracellular fatty acids, oxidized lipids and cholesterol [42, 62, 63]. The tumor microenvironment also enhances ACC activity to promote lipid biogenesis and storage. Inhibition of the ACC in CD8+ T cells facilitates FAO and decreases lipid accumulation, thereby restoring their effector function [50]. In intrapancreatic CD8+ T cells, lipid accumulation impairs mitochondrial function, but enforced expression of the very-long-chain acyl-CoA dehydrogenase enzyme (ACADVL) can rescue mitochondrial function and protect T cells from lipotoxicity, thereby increasing intratumor T-cell survival and persistence [82].

### Shifts in energy metabolism

Lipid accumulation in CD8+ T cells causes a shift in energy metabolism. Tumor-infiltrating exhausted CD8+ T cells rely more on FAO for energy than on glycolysis and OXPHOS. Therefore, maintaining FAO activity in CD8+ T cells is crucial for controlling tumor growth. In a melanoma model, CD8+ TILs enhanced PPARα signaling and fatty acid catabolism, partially preserving their effector functions. Promoting FAO with a PPARα agonist further improved the ability of CD8+ TILs to slow tumor progression [47]. The STAT3-CPT1A axis is an important FAO regulator, but its role in tumor-infiltrating CD8+ T-cell function is unclear. IL-9-mediated STAT3 activation increases FAO in tumor Tc9 cells, increasing their longevity and antitumor ability. IL-9 signaling deficiency, STAT3 inhibition, or increased lipid peroxidation result in impaired Tc9 function [75]. Conversely, in obesity tumor models, activated STAT3 promotes FAO while inhibiting glycolysis and IFNγ production in CD8+ effector T cells. Genetic ablation of STAT3 in T cells or CPT1A inhibitor treatment in obese tumor-bearing mice suppresses breast tumor progression by reducing FAO, increasing glycolysis, and enhancing CD8+ T-cell functions [74].

### Disruptions in mitochondrial dynamics

Mitochondrial dynamics encompass fusion and fission processes, as well as mitochondrial mobility, biogenesis and autophagy. Disordered mitochondrial dynamics can affect CD8+ T-cell function. Tumor-infiltrating CD8+ T cells exhibit decreased mitochondrial function and mass. Proliferator-activated receptor gamma coactivator 1-alpha (PGC-1α), the master regulator of mitochondrial biogenesis, is progressively lost in tumor-infiltrating CD8+ T cells. Akt-mediated inhibition of PGC-1α represses T-cell mitochondrial biogenesis [49]. Forced PGC-1α expression maintains increased mitochondrial activity and promotes CD8+ T-cell fitness, memory formation and antitumor immunity [109]. Moreover, rapamycin-treated human CAR-T cells exhibit superior mitochondrial metabolism and intertumoral motility in 3D spheroid assays and multiple solid tumor models [110]. These results reinforce the important role of mitochondria in maintaining antitumor activity in CD8+ T cells. Furthermore, mitochondria‒endoplasmic reticulum contact sites (MERCs) are highly structured regions that control lipid homeostasis and mitochondrial metabolism [111]. Compared with those of effector T cells, enhanced MERCs have been observed in memory T cells [112]. Linoleic acid treatment promotes the formation of MERCs, enhancing mitochondrial energetics and CD8+ T-cell effector function [85]. The profound effects of MERCs on tumor-infiltrating CD8+ T cells require further investigation.

### Increased ROS production and cell death

Oxidative stress is tightly regulated by mitochondria in CD8+ T cells through development and activation. In the lipid overload tumor microenvironment, this equilibrium is skewed toward an oxidative state via lipid peroxidation and the formation of reactive lipid aldehydes. Increased lipid uptake in tumor-infiltrating CD8+ T cells promotes lipid peroxidation, inducing ROS-mediated apoptosis and ferroptosis [113]. High ROS levels are major factors that inhibit T-cell activation and proliferation in the tumor microenvironment [114]. To increase inherent resistance to oxidative stress in tumors, CD8+ T cells activate antioxidant systems (glutathione and thioredoxin systems) to maintain oxidative balance [115]. ROS scavengers can restore CD8+ TIL activation in human renal cell tumors by activating mitochondrial superoxide dismutase 2 (SOD2) [116]. Therefore, reducing lipid uptake or using cell death inhibitors can enhance CD8+ T-cell function and suppress tumor growth [41, 56, 75, 84]. Moreover, pharmacological activation of nuclear factor E2-related factor 2 (Nrf2) by auranofin can restore the activity of TILs, including NK cells, T cells, and engineered CAR-T cells, even under conditions of high ROS stress [117].

In summary, the lipid-enriched TME significantly impacts mitochondrial function in CD8+ T cells, affecting their metabolism, dynamics, and survival. Understanding these interactions is important for developing strategies to increase the efficacy of CD8+ T cells in antitumor immunity.

## Effects of systemic lipid metabolism changes on CD8+ T cells

**Fig. 4:**
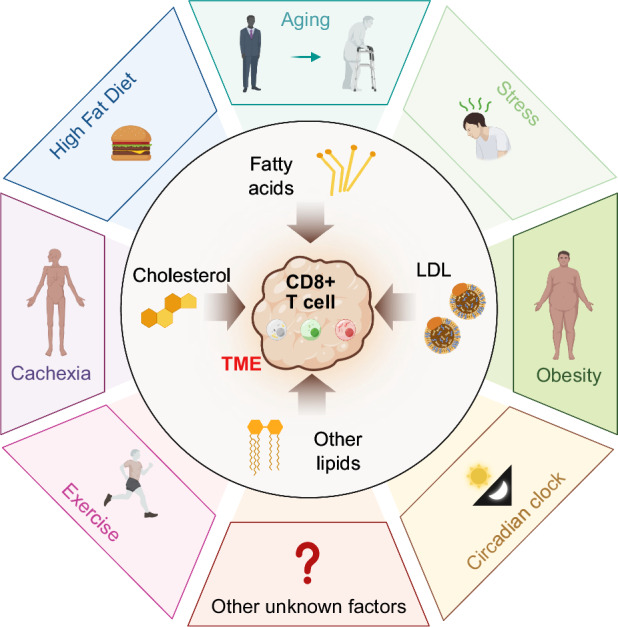
Systemic factors regulating lipid metabolism and antitumor immunity. This figure represents several systemic factors that regulate lipid metabolism and influence CD8+ T-cell-mediated antitumor immunity, including a high-fat diet, obesity, aging, cachexia, circadian rhythms, stress, exercise, and other unknown factors. These factors modulate lipid metabolism in the body, thereby affecting the immune response to tumors

The nutrients supplied by the host influence tumor cell growth and survival. Therefore, systemic metabolic changes in the body modulate tumor progression and metastasis. Clinical observations have indicated that the nutritional or metabolic state of patients is correlated with cancer therapeutic efficacy. The metabolic state can be altered by many environmental factors, such as obesity, aging, diet, and stress. This section discusses how systemic lipid metabolism changes impact tumor progression, especially by affecting the function of tumor-infiltrating CD8+ T cells (Fig. 4).

### Obesity and high-fat diet

Obesity is not merely an issue of excess body fat or being overweight; it is a complex medical condition that significantly elevates the risk of numerous other diseases and health complications. According to reports from the World Health Organization, in 2016, there were over 650 million obese adults globally, with 36% of U.S. adults classified as obese [118]. Obesity drives systemic perturbations, including the expansion of white adipose tissue (WAT), dyslipidemia, hypercholesterolemia, insulin resistance, altered hormones, and inflammatory changes. Dysfunction of these metabolic processes can affect tumor initiation, progression and metastasis [119]. Epidemiological evidence has established a strong link between obesity and an increased risk of cancer development, as well as adverse outcomes and poorer prognoses in many cancer types, such as breast cancer and liver cancer [120, 121]. However, the impact of systemic metabolic changes associated with obesity on antitumor immunity within the local tumor microenvironment remains poorly understood.

Studies using HFD-induced obesity models have indicated that obesity can induce an immunosuppressive tumor microenvironment characterized by the accumulation of myeloid-derived suppressor cells (MDSCs) [122], macrophages [123, 124] and neutrophils [125, 126] and the dysfunction of dendritic cells [127], natural killer (NK) cells [128] and T cells [74, 129, 130]. In MC38 (colorectal cancer), EO771 (breast cancer), and B16 (melanoma) but not LLC (lung cancer) tumor models, HFD-induced obesity accelerated tumor growth in a CD8+ T-cell-dependent manner. Single-cell RNA sequencing (scRNA-seq) analysis revealed that tumor-infiltrating CD8+ T cells from HFD-fed tumors presented enriched lipid metabolic signatures, exclusion from GLUT1-high regions, and increased expression of exhaustion markers. Interestingly, a HFD causes opposing metabolic changes in CD8+ T cells and tumor cells. A HFD promotes fatty acid uptake in tumor cells, while tumor-infiltrating CD8+ T cells cannot efficiently take up fatty acids, resulting in impaired effector function [129]. In a related study using the PyMT breast tumor model, which develops tumors in the lipid-rich environment of the mammary gland, obesity impaired CD8+ T-cell function by upregulating FAO and inhibiting glycolysis [74]. Obesity can also induce exhaustion of adipose tissue-residential T cells [131]. However, whether obesity affects the function of adipose tissue-residential CD8+ T cells, thereby impacting mammary tumor growth, remains to be further investigated. Moreover, obesity decreases the expression of CXCR3 and CD49d, which are crucial for attracting T cells to tumor sites, resulting in reduced T-cell infiltration in obese tumors [130].

The composition of dietary fats can influence CD8+ T-cell-mediated antitumor immunity. For example, a diet enriched with saturated fatty acids promotes mammary tumor cell growth, whereas a diet rich in omega-3 polyunsaturated fatty acids does not impact tumor growth. A mechanistic study indicated that omega-3 fatty acids increase ROS production and induce apoptosis in macrophages [92]. As omega-3 fatty acids have been shown to affect CD8+ T-cell function in other studies [132, 133], their effects on tumor-infiltrating CD8+ T cells need further elucidation. Additionally, a diet enriched with linoleic acid, but not oleic acid, promotes mammary tumor growth by dampening CD8+ T-cell function and inducing apoptosis [84]. This evidence suggests that being overweight is not the sole factor driving tumor progression in obese individuals; rather, the composition and levels of specific lipids may be more critical. Determining which lipids are healthier than others is of great interest, as these lipids could serve as diagnostic factors or predictors for the efficacy of ICB therapy. The most recent American Dietary Guidelines (2020–2025) recommend shifting dietary fats from saturated to unsaturated [84], and incorporating specific fats into the diet may serve as an adjuvant for cancer treatment.

Glucagon-like peptide-1 receptor (GLP-1R) agonists are a class of drugs that target incretin hormone action and have shown great efficacy in treating obesity by promoting weight loss [134]. However, semaglutide, an FDA-approved GLP-1R agonist, does not improve antitumor immunity in obese mice, although it significantly induces weight loss. In contrast, diet restriction-induced weight loss can rescue CD8+ TIL effector function [135]. Recent studies have shown that GLP-1R is expressed in CD8+ T cells and acts as a T-cell-negative costimulatory molecule. GLP-1R antagonism enhances antitumor immunity, inhibiting colorectal tumor growth [136]. Investigating the mechanism behind the different effects of diet restriction and GLP-1R agonist/antagonist treatment in tumor models would be an interesting direction for future investigations.

Although obesity has been shown to suppress antitumor immunity in both animal mouse models and clinical observations [119, 137], paradoxically, it increases the efficacy of PD-1/PD-L1 blockade therapies [135, 138]. ICB reprograms immunosuppressive splenic immune cells induced by obesity, and a potential microbial signature has been identified as a biomarker for ICB therapeutic efficacy in obesity [124]. Leptin, which is present at high levels in obese individuals, is positively correlated with PD-1 expression in CD8+ T cells in obese individuals. High leptin levels in obese mice drive PD-1-mediated T-cell exhaustion in melanoma, and a lack of leptin signaling restores T-cell function [138]. A previous meta-analysis also suggested that leptin levels could serve as a diagnostic tool for breast cancer [139]. However, recent studies suggest that obesity selectively induces PD-1 expression in tumor-associated macrophages. PD-1 expression in these macrophages is induced by obesity-related factors, including IFNγ, TNF, leptin, insulin and palmitate. PD-1 signaling in macrophages suppresses glycolysis, phagocytosis, and T-cell stimulatory potential [140]. A deeper investigation into the factors influencing the obesity paradox will enhance the application of ICB therapies.

### Aging and other systemic factors

Cancer is an age-related disease, with most cancers occurring in elderly individuals. Aging involves widespread metabolic changes and is usually accompanied by harmful lipid storage and peroxidation [141]. One reason for dysregulated lipid metabolism in aging is decreased mitochondrial function [142]. T-cell-mediated immune responses are compromised in aged individuals, with aged CD4+ T cells generating smaller mitochondria with lower respiratory capacity and defective one-carbon metabolism. Supplementation with one-carbon metabolites can restore aged T-cell activation and survival [143]. Interestingly, ICB therapies exhibit great efficacy in some aged animal tumor models and human cancer patients. Responsiveness to anti-PD-1 therapy is increased in aged human melanoma patients and aged mice and is associated with increased CD8+ T-cell infiltration [144]. Senescence is a cellular response implicated in age-related disease [145]. T-cell senescence in tumor cells is a general feature of cancers. Senescent T cells display unbalanced lipid metabolism, leading to alterations in lipid species and the accumulation of lipid droplets in CD8+ T cells. Reprogramming lipid metabolism can prevent T-cell senescence and enhance antitumor immunity and immunotherapy efficacy in melanoma and breast cancers [146]. Further investigations are needed to explore the relationships among aging, lipid metabolism, and CD8+ T cells within the tumor context.

Cachexia is a wasting syndrome characterized by increased catabolism, weight loss, decreased skeletal muscle mass, and inflammation. Cachexia commonly occurs in various cancers and significantly contributes to cancer-related mortality [147]. In cancer-associated cachexia (CAC), increased catabolism of white adipose tissue (WAT) leads to lipotoxicity and an increase in circulating free fatty acids. In addition, reduced lipid production and the interaction between adipose tissue and the immune system aggravate the progression of CAC [148]. In patients with CAC, circulating levels of recent thymic migrants and effector memory CD8+ T cells are associated with increased muscle mass [149], suggesting that an efficient antitumor CD8+ T-cell response may protect against muscle catabolism. CD8+ T cells modulate adipose tissue lipid metabolism during chronic viral infection in a type I IFN-dependent manner, with mice lacking CD8+ T cells showing minimal weight loss following infection [150].

Systemic metabolism can also be influenced by exercise, stress and the circadian clock. Aerobic exercise training reduces the incidence and mortality of several cancer types. Multiple studies in animal models have shown that proper excision can restrict tumor growth by increasing CD8+ T-cell-mediated antitumor immunity. Exercise training changes the metabolites in the body and alters the effector function of CD8+ T cells [151]. Moderate-intensity aerobic exercise training can reduce mitochondrial loss, thereby improving the effector function of CD8+ T cells [152]. Moreover, exercise training sensitized ICB-refractory breast cancer patients to treatment [153].

Psychological stress affects inflammation and metabolism [154], promoting cancer progression and metastasis [155]. Chronic stress increases lung metastasis in patients with breast cancer by modulating neutrophil function [156]. Stress also impairs vaccine-specific CD8+ T-cell generation and tumor protection [157]. Stress-associated catecholamines promote T-cell exhaustion through β1-adrenergic receptor signaling, and blocking this pathway can increase the efficacy of ICB therapy [158]. The circadian clock regulates daily physiological rhythms, with circadian disruption enhancing tumor formation and metastasis [159]. Fatty acid oxidation senses circadian disruption in sleep deficiency-enhanced tumorigenesis, with ACSL1 expression in tumors promoting lung tumorigenesis [160]. CD8+ TILs are also regulated by the circadian clock, and timing of treatment during the day can improve CAR-T-cell and ICB therapy efficacies [161].

In summary, systemic factors such as obesity, aging, and other metabolic changes significantly influence lipid metabolism and CD8+ T-cell function in the TME. Exploring these interactions could open new avenues for developing more effective cancer treatments.

## Reprogramming lipid metabolism in CD8+ T cells to improve immunotherapy

Immunotherapy has achieved remarkable advancements in cancer treatment. However, owing to the complexity of the tumor microenvironment and macroenvironment, its efficacy remains limited in most solid tumors, especially metastatic ones [3]. Activated T cells with PD-1 ligation increase the FAO of endogenous lipids but are unable to engage in glycolysis or amino acid metabolism. In contrast, CTLA-4 signaling inhibits glycolytic reprogramming without increasing FAO [162]. These findings indicate that metabolic regulation plays a crucial role in the PD-1-mediated blockade of T-effector cell differentiation. Furthermore, Tpex cells, known for their metabolic fitness, are key players in generating effector T cells during anti-PD-1 therapy by undergoing a proliferative burst. Thus, rewiring lipid metabolism in tumor CD8+ T cells could be a promising therapeutic strategy to increase immunotherapy efficacy (Table 1).

**Table 1:** Targetting lipid metabolism in CD8+ T cells to improve antitumor immunity and immunotherapy efficacy

Targets	Drug	Combined immunotherapy	Cancer types	Working mechanisms	Effects on CD8+ T cells	References
CD36	CD36 mAb	NO	Melanoma(B16F10), Colorectal cancer(MC38)	Decrease uptake of lipids and reduce ferroptosis	Restore effector function	[163]
Akt	Akti-1/2	Adoptive cell transfer	Melanoma(B16F10)	Ehance FAO	Enable expansion and enhance persistence in vivo	[165]
PPAR	Fenofibrate	Adoptive cell transfer and Anti-PD-1	Melanoma(B16-Ova)	Ehance FAO	Improve effector function	[47]
	Bezafibrate	Anti-PD-L1	Colorectal cancer(MC38), Skin sacroma (MethA)	Activare PGC-1α/PPAR and ehance FAO	Increase proliferation of naïve T cells and improve effector function	[48, 71]
	GW501516	Adoptive cell transfer	Melanoma(B16F10-gp33)	Increase CPT1A expression and ehance FAO	Enhance persistence in vivo	[166]
CPT1	Etomoxir and Pherhexiline	NO (High fat diet model)	Breast (PyMT tumor mice)	Inhibit FAO but promote glycolysis	Restore effector function	[74]
HMGCR	Statin	Anti-PD-1	Lewis lung cancer cells (NSCLC)	Inhibit PD-L1expression in tumor cells	Increase T cell infiltrations	[167]
PCSK9	PF-06446846 or Neutralizing antibody	Anti-PD-1	Melanoma(B16F10), Colorectal cancer(MC38)	Disrupt the recycling of MHC I on tumors and modulate TCR signal	Restore effector function	[63, 94]
ACAT1	Avasimibe	Anti-PD-1	Melanoma(B16F10)	Enhance TCR clustering and signaling	Potentiate effector function	[96]
Mevalonate pathway	Lipophilic statins and Lipophilic bisphosphonates	Anti-PD-1	Melanoma(B16-Ova), Lung cancer (TC-1)	Increase antigen preservation and presentation	Induces strong cytotoxic T lymphocyte response	[170]
Ferroptosis pathway	Cyst(e)inase	Anti-PD-L1	Ovarian cancer(ID8)	Induce tumor cell ferropototis with IFNγ	Boost T cell cytotoxicity	[172]
	Arachidonic acid	Anti-PD-L1	Colorectal cancer(MC38), Melanoma(Yumm5.2)	Induce tumor cell ferropototis with IFNγ	Boost T cell cytotoxicity	[87]
	Ferrostatin-1	Adoptive cell transfer	Melanoma(B16F10)	Inhibit T cell ferropototis	Restore effector function	[41]

Lipid uptake from the tumor environment is crucial for maintaining CD8+ T-cell effector function, as tumors often lack glucose as an energy source. However, excessive lipid uptake can result in lipotoxicity and trigger ferroptosis in tumor-infiltrating T cells. The lipid transporter CD36 is highly expressed in tumor CD8+ T cells, impairing their function. Genetic knockout of CD36 in CD8+ T cells significantly suppresses tumor growth in colorectal cancer and melanoma [41, 42]. The use of an anti-CD36-blocking antibody alone restores CD8+ T-cell function and inhibits melanoma growth [42]. Interestingly, an anti-CD36-blocking antibody can also target metastasis-initiating cells to inhibit metastasis in immunodeficient or immunocompetent mouse models without side effects [163]. Consequently, CD36 presents a promising drug target, and the development of novel CD36 inhibitors, especially in combination with immunotherapy, holds significant potential for advancing cancer treatment.

Compared with other cell states, tumor CD8+ T cells are highly dependent on FAO for energy generation. Enhancing the FAO pathway could increase the cytotoxicity of CD8+ T cells in tumors. The serine/threonine kinase Akt is involved in regulating CD8+ T-cell differentiation and memory formation by modulating metabolic pathways [164]. Akt inhibition is associated with increased FAO in human TILs. In ACT therapy, pharmacologic inhibition of Akt enables the expansion of TILs and enhances T-cell persistence in vivo [165]. The PPAR family of transcription factors broadly regulates FAO. In an ACT model, CD8+ T cells treated with the PPARα agonist fenofibrate improved CD8+ TIL function and synergized with PD-1 blockade to delay tumor growth [47]. Bezafibrate enhances mitochondrial function by activating the PGC-1a/PPAR complex. Combining Bezafibrate with anti-PD-L1 enhances the survival and proliferation of tumor-reactive cytotoxic CD8+ T cells [48, 71]. GW501516, a PPARδ agonist that also activates PPARα/β, increases FAO in CD8+ T cells, increasing IFNγ production and T-bet expression. In ACT mouse models, GW501516-treated CD8+ T cells show enhanced persistence in vivo and superior tumor-killing efficacy [166]. However, overactivation of FAO in CD8+ T cells in HFD-induced obese models contributes to tumor promotion. Targeting CPT1 with etomoxir or perhexiline to inhibit FAO can restore effector T-cell function and restrict tumor growth [74]. Given the strong efficacy of anti-PD-1 therapy in obese tumor models [138], testing the combination of CPT1 inhibitors and anti-PD-1 therapy in these models would be interesting.

Abnormal cholesterol synthesis is closely related to tumor development, growth, and progression. Most therapeutic targets for cholesterol metabolism focus on tumor cells. Statins, which selectively inhibit HMG-CoA reductase, a rate-limiting enzyme in the mevalonate biosynthesis pathway, suppress non-small lung cancer (NSCLC) progression and shape an inflamed tumor microenvironment. Statins can transcriptionally inhibit PD-L1 expression in tumor cells and enhance the response to ICB therapy [167]. Statins have also shown efficacy in improving ICB therapies in advanced renal cell carcinoma and head and neck cancer [168, 169]. PCSK9 has emerged as an attractive target for cancer therapy, as its expression in both tumor cells and CD8+ T cells can suppress tumor progression by modulating cholesterol metabolism. Treatment with the PCSK9 inhibitor PF-06446846 can effectively potentiate the antitumor immunity of CD8+ T cells and suppress tumor growth [63]. Clinically approved PCSK9-neutralizing antibodies synergize with anti-PD-1 therapy to inhibit tumor growth [94]. Avasimibe, an ACAT inhibitor previously used as a lipid-lowering agent for atherosclerosis treatment, shows better therapeutic efficacy in controlling tumor progression when combined with an anti-PD-1 antibody [96]. The mevalonate (MVA) pathway, which controls cholesterol biosynthesis, is a core metabolic pathway and a druggable target for vaccine adjuvant discovery. In multiple mouse models, MVA pathway inhibitors, such as lipophilic statins and lipophilic bisphosphonates, have demonstrated robustness in cancer vaccinations and synergy with anti-PD-1 antibodies [170]. Targeting various aspects of cholesterol metabolism is highly important for increasing antitumor immunity.

Ferroptosis, a type of programmed cell death characterized by iron accumulation and lipid peroxidation [171], is influenced by altered lipid metabolism in the TME. IFNγ released by immunotherapy-activated CD8+ T cells downregulates SLC3A2 and SLC7A11 expression, enhancing ferroptosis-specific lipid peroxidation in tumor cells. Cyst(e)inase, an engineered enzyme that induces oxidative stress and promotes ferroptosis, synergizes with anti-PD-L1 treatment to increase antitumor immunity [172]. Subsequent studies indicated that IFNγ and arachidonic acid from the tumor microenvironment jointly induce tumor cell ferroptosis through ACSL4. Thus, IFNγ signaling paired with selective fatty acids naturally facilitates CD8+ T-cell-induced tumor killing. Arachidonic acid treatment or genetic deletion of ACSL4 sensitizes tumors to PD-1/PD-L1 blockade treatment [87]. Ferroptosis inducers can elicit ferroptosis not only in tumor cells but also in tumor CD8+ T cells. Pharmacologic screening has shown that CD8+ T cells are more sensitive to ferroptosis inducers than B16 and MC38 cancer cells, but inhibiting ferroptosis in CD8+ T cells impairs the antitumor response [173]. Paradoxically, another study revealed that CD36-mediated ferroptosis dampens intratumor CD8+ T-cell effector function and impairs antitumor immunity. In adoptive T-cell transfer models, CD8+ T cells treated with the ferroptosis inhibitor ferrostatin-1 can suppress tumor growth, whereas T cells treated with the ferroptosis inducer RSL-3 promote tumor growth [41]. Similarly, ferrostatin-1–treated Tc9 cells display reduced iron levels and lipid peroxidation, resulting in increased antitumor ability in vivo [75]. Overall, targeting ferroptosis via different strategies can augment antitumor immunity and control tumor growth.

## Concluding remarks and future directions

Cancer is widely recognized as a systemic disease [2]. Tumor cells obtain and utilize nutrients from nutrient-poor microenvironments to meet their demands for survival and growth. Moreover, the nutrient-poor microenvironment also creates an immunosuppressive environment that allows tumor cells to escape immune surveillance. Recent studies have indicated that lipids are abundant in tumor interstitial fluids and have emerged as suppressors of antitumor immunity [174]. Factors causing systemic lipid metabolism changes may impact antitumor immunity.

Dietary interventions have been proven to be efficient in controlling tumor growth. For example, low-glycemic diets can alter lipid metabolism to suppress pancreatic cancer growth [175]. Clinically, fasting-mimicking diets have shown a complete or partial response to neoadjuvant chemotherapy in breast cancer patients [176]. Although some lipid-containing diets have shown efficacy in facilitating CD8+ T-cell-mediated antitumor responses, the impact of dietary restriction of specific lipids on CD8+ T-cell antitumor function remains unknown. Moreover, systemic lipid metabolism changes affect tumor growth, and tumors can conversely influence systemic lipid metabolism. For example, distant tumor cells secrete extracellular vesicles and particles containing fatty acids, generating a proinflammatory microenvironment, promoting fatty liver formation, and limiting chemotherapy tolerance [177]. In another study, HFD-induced fatty liver increased the production of hepatocyte-derived extracellular vesicles, promoting a metastatic tumor microenvironment [178]. Since the liver is the major organ regulating systemic lipid metabolism, studying the metabolic interaction between the liver and distant tumors is highly important. Lipid metabolism dysfunction in the liver may also influence CD8+ T-cell function in tumor progression. Another important aspect is the molecular mechanisms by which lipids impact mitochondrial function in tumor-infiltrating CD8+ T cells. Targeting mitochondria to restore CD8+ T-cell function has been shown to increase the efficacy of immunotherapy [71, 166, 179]. Moreover, a high OXPHOS CD8+ T-cell subset is predictive of immunotherapy resistance in melanoma patients [180].

As CD8+ T cells at different stages exhibit distinct metabolic characteristics, careful evaluation of the role of specific lipids in tumor-infiltrating CD8+ T cells within various contexts is crucial. Identifying key genes involved in specific lipid metabolism and using genetically modified mice as tools will be very helpful for further studies. Advanced mass spectrometry technology is essential for identifying novel lipids involved in the regulation of tumor-infiltrating CD8+ T cells. The development of novel lipid tracers for isotopic labeling would provide valuable information on lipid metabolic fluxes in vivo. Matrix-assisted laser desorption/ionization (MALDI) is a mass spectrometry-based technique that can detect tissue metabolites in situ. Combining MALDI with immunofluorescence staining has shown that long-chain fatty acids are enriched in tumors and drive CD8+ T-cell dysfunction [82]. Recently, integrating MALDI imaging with spatial transcriptomics has been used to visualize intratumor metabolic heterogeneity and cell metabolic interactions in the same gastric cancer sample [181]. The application of spatial omics would provide spatiotemporal information on lipid metabolism and its impacts on tumor-infiltrating CD8+ T cells. Overall, the development of new methods and the application of novel technologies are urgently needed to better understand lipid metabolism regulation in tumor-infiltrating CD8+ T cells.

In this review, we comprehensively review the current knowledge concerning lipid metabolism regulation in tumor-infiltrating CD8+ T cells. Moreover, we speculated that tumors are systemic diseases that can be modulated by systemic factors that are involved in lipid metabolism regulation. These new findings highlight lipids as promising targets for enhancing the effectiveness of immunotherapy. However, more detailed studies on the regulation of lipid metabolism in tumor-infiltrating CD8+ T cells at both the cellular and genetic levels are necessary to expedite clinical applications.

## References

[1] Mattiuzzi C, Lippi G. Current Cancer Epidemiology. J Epidemiol Glob Health. 2019;9:217–22. 10.2991/jegh.k.191008.001. 31854162 10.2991/jegh.k.191008.001 PMC7310786

[2] Swanton C, Bernard E, Abbosh C, André F, Auwerx J, Balmain A, et al. Embracing cancer complexity: Hallmarks of systemic disease. Cell. 2024;187:1589–616. 10.1016/j.cell.2024.02.009. 38552609 10.1016/j.cell.2024.02.009 PMC12077170

[3] Esposito M, Ganesan S, Kang Y. Emerging strategies for treating metastasis. Nat Cancer. 2021;2:258–70. 10.1038/s43018-021-00181-0. 33899000 10.1038/s43018-021-00181-0 PMC8064405

[4] Hanahan D. Hallmarks of Cancer: New Dimensions. Cancer Discov. 2022;12:31–46. 10.1158/2159-8290.CD-21-1059. 35022204 10.1158/2159-8290.CD-21-1059

[5] Cockcroft S. Mammalian lipids: structure, synthesis and function. Essays Biochem. 2021;65:813–45. 10.1042/EBC20200067. 34415021 10.1042/EBC20200067 PMC8578989

[6] Hodson L, Gunn PJ. The regulation of hepatic fatty acid synthesis and partitioning: the effect of nutritional state. Nat Rev Endocrinol. 2019;15:689–700. 10.1038/s41574-019-0256-9. 31554932 10.1038/s41574-019-0256-9

[7] Zhang W, Xu L, Zhu L, Liu Y, Yang S, Zhao M. Lipid Droplets, the Central Hub Integrating Cell Metabolism and the Immune System. Front Physiol. 2021;12:746749. 10.3389/fphys.2021.746749. 34925055 10.3389/fphys.2021.746749 PMC8678573

[8] Ikonen E, Zhou X. Cholesterol transport between cellular membranes: A balancing act between interconnected lipid fluxes. Dev Cell. 2021;56:1430–6. 10.1016/j.devcel.2021.04.025. 34004151 10.1016/j.devcel.2021.04.025

[9] Song Y, Liu J, Zhao K, Gao L, Zhao J. Cholesterol-induced toxicity: An integrated view of the role of cholesterol in multiple diseases. Cell Metab. 2021;33:1911–25. 10.1016/j.cmet.2021.09.001. 34562355 10.1016/j.cmet.2021.09.001

[10] Jain A, Zoncu R. Organelle transporters and interorganelle communication as drivers of metabolic regulation and cellular homeostasis. Mol Metab. 2022;60:101481. 10.1016/j.molmet.2022.101481. 35342037 10.1016/j.molmet.2022.101481 PMC9043965

[11] Rosen ED, Spiegelman BM. Molecular regulation of adipogenesis. Annu Rev Cell Dev Biol. 2000;16:145–71. 10.1146/annurev.cellbio.16.1.145. 11031233 10.1146/annurev.cellbio.16.1.145

[12] Morigny P, Boucher J, Arner P, Langin D. Lipid and glucose metabolism in white adipocytes: pathways, dysfunction and therapeutics. Nat Rev Endocrinol. 2021;17:276–95. 10.1038/s41574-021-00471-8. 33627836 10.1038/s41574-021-00471-8

[13] Song Z, Xiaoli AM, Yang F. Regulation and Metabolic Significance of De Novo Lipogenesis in Adipose Tissues. Nutrients. 2018;10:1383. 10.3390/nu10101383. 30274245 10.3390/nu10101383 PMC6213738

[14] Jones JG. Hepatic glucose and lipid metabolism. Diabetologia. 2016;59:1098–103. 10.1007/s00125-016-3940-5. 27048250 10.1007/s00125-016-3940-5

[15] Sakers A, De Siqueira MK, Seale P, Villanueva CJ. Adipose-tissue plasticity in health and disease. Cell. 2022;185:419–46. 10.1016/j.cell.2021.12.016. 35120662 10.1016/j.cell.2021.12.016 PMC11152570

[16] Kersten S, Seydoux J, Peters JM, Gonzalez FJ, Desvergne B, Wahli W. Peroxisome proliferator-activated receptor alpha mediates the adaptive response to fasting. J Clin Invest. 1999;103:1489–98. 10.1172/JCI6223. 10359558 10.1172/JCI6223 PMC408372

[17] Qian X, Yang Z, Mao E, Chen E. Regulation of fatty acid synthesis in immune cells. Scand J Immunol. 2018;88:e12713. 10.1111/sji.12713. 30176060 10.1111/sji.12713

[18] Kohli GS, John U, Van Dolah FM, Murray SA. Evolutionary distinctiveness of fatty acid and polyketide synthesis in eukaryotes. ISME J. 2016;10:1877–90. 10.1038/ismej.2015.263. 26784357 10.1038/ismej.2015.263 PMC5029157

[19] Stark JM, Tibbitt CA, Coquet JM. The Metabolic Requirements of Th2 Cell Differentiation. Front Immunol. 2019;10:2318. 10.3389/fimmu.2019.02318. 31611881 10.3389/fimmu.2019.02318 PMC6776632

[20] Lee J, Walsh MC, Hoehn KL, James DE, Wherry EJ, Choi Y. Regulator of fatty acid metabolism, acetyl coenzyme a carboxylase 1, controls T-cell immunity. J Immunol. 2014;192:3190–9. 10.4049/jimmunol.1302985. 24567531 10.4049/jimmunol.1302985 PMC3965631

[21] Kidani Y, Elsaesser H, Hock MB, Vergnes L, Williams KJ, Argus JP, et al. Sterol regulatory element-binding proteins are essential for the metabolic programming of effector T cells and adaptive immunity. Nat Immunol. 2013;14:489–99. 10.1038/ni.2570. 23563690 10.1038/ni.2570 PMC3652626

[22] Su W, Chapman NM, Wei J, Zeng H, Dhungana Y, Shi H, et al. Protein Prenylation Drives Discrete Signaling Programs for the Differentiation and Maintenance of Effector T(reg) Cells. Cell Metab. 2020;32:996–1011. 10.1016/j.cmet.2020.10.022. 33207246 10.1016/j.cmet.2020.10.022 PMC7887758

[23] Porstmann T, Griffiths B, Chung YL, Delpuech O, Griffiths JR, Downward J, et al. PKB/Akt induces transcription of enzymes involved in cholesterol and fatty acid biosynthesis via activation of SREBP. Oncogene. 2005;24:6465–81. 10.1038/sj.onc.1208802. 16007182 10.1038/sj.onc.1208802

[24] Luo J, Yang H, Song BL. Mechanisms and regulation of cholesterol homeostasis. Nat Rev Mol Cell Biol. 2020;21:225–45. 10.1038/s41580-019-0190-7. 31848472 10.1038/s41580-019-0190-7

[25] Bao C, Wu T, Zhu S, Wang X, Zhang Y, Wang X, et al. Regulation of cholesterol homeostasis in osteoporosis mechanisms and therapeutics. Clin Sci. 2023;137:1131–43. 10.1042/CS20220752. 10.1042/CS20220752 37553962

[26] Bartlett K, Eaton S. Mitochondrial beta-oxidation. Eur J Biochem. 2004;271:462–9. 10.1046/j.1432-1033.2003.03947.x. 14728673 10.1046/j.1432-1033.2003.03947.x

[27] Su X, Abumrad NA. Cellular fatty acid uptake: a pathway under construction. Trends Endocrinol Metab. 2009;20:72–77. 10.1016/j.tem.2008.11.001. 19185504 10.1016/j.tem.2008.11.001 PMC2845711

[28] Senzaki H, Iwamoto S, Ogura E, Kiyozuka Y, Arita S, Kurebayashi J, et al. Dietary effects of fatty acids on growth and metastasis of KPL-1 human breast cancer cells in vivo and in vitro. Anticancer Res. 1998;18:1621–7. 9673380

[29] Bani IA, Williams CM, Boulter PS, Dickerson JW. Plasma lipids and prolactin in patients with breast cancer. Br J Cancer. 1986;54:439–46. 10.1038/bjc.1986.195. 3756079 10.1038/bjc.1986.195 PMC2001617

[30] Madak-Erdogan Z, Band S, Zhao YC, Smith BP, Kulkoyluoglu-Cotul E, Zuo Q, et al. Free Fatty Acids Rewire Cancer Metabolism in Obesity-Associated Breast Cancer via Estrogen Receptor and mTOR Signaling. Cancer Res. 2019;79:2494–510. 10.1158/0008-5472.CAN-18-2849. 30862719 10.1158/0008-5472.CAN-18-2849

[31] Zuo Q, Band S, Kesavadas M, Madak Erdogan Z. Obesity and Postmenopausal Hormone Receptor-positive Breast Cancer: Epidemiology and Mechanisms. Endocrinology. 2021;162, 10.1210/endocr/bqab195. 10.1210/endocr/bqab195 34519778

[32] Calderon-Dominguez M, Sebastián D, Fucho R, Weber M, Mir JF, García-Casarrubios E, et al. Carnitine Palmitoyltransferase 1 Increases Lipolysis, UCP1 Protein Expression and Mitochondrial Activity in Brown Adipocytes. PLoS One. 2016;11:e0159399. 10.1371/journal.pone.0159399. 27438137 10.1371/journal.pone.0159399 PMC4954705

[33] Houten SM, Wanders RJ. A general introduction to the biochemistry of mitochondrial fatty acid beta-oxidation. J Inherit Metab Dis. 2010;33:469–77. 10.1007/s10545-010-9061-2. 20195903 10.1007/s10545-010-9061-2 PMC2950079

[34] Kerner J, Hoppel C. Fatty acid import into mitochondria. Biochim Biophys Acta. 2000;1486:1–17. 10.1016/s1388-1981(00)00044-5. 10856709 10.1016/s1388-1981(00)00044-5

[35] Houten SM, Violante S, Ventura FV, Wanders RJ. The Biochemistry and Physiology of Mitochondrial Fatty Acid beta-Oxidation and Its Genetic Disorders. Annu Rev Physiol. 2016;78:23–44. 10.1146/annurev-physiol-021115-105045. 26474213 10.1146/annurev-physiol-021115-105045

[36] Swigonova Z, Mohsen AW, Vockley J. Acyl-CoA dehydrogenases: Dynamic history of protein family evolution. J Mol Evol. 2009;69:176–93. 10.1007/s00239-009-9263-0. 19639238 10.1007/s00239-009-9263-0 PMC4136416

[37] Aoyama T, Peters JM, Iritani N, Nakajima T, Furihata K, Hashimoto T, et al. Altered constitutive expression of fatty acid-metabolizing enzymes in mice lacking the peroxisome proliferator-activated receptor alpha (PPARalpha). J Biol Chem. 1998;273:5678–84. 10.1074/jbc.273.10.5678. 9488698 10.1074/jbc.273.10.5678

[38] Lee SS, Pineau T, Drago J, Lee EJ, Owens JW, Kroetz DL, et al. Targeted disruption of the alpha isoform of the peroxisome proliferator-activated receptor gene in mice results in abolishment of the pleiotropic effects of peroxisome proliferators. Mol Cell Biol. 1995;15:3012–22. 10.1128/MCB.15.6.3012. 7539101 10.1128/mcb.15.6.3012 PMC230532

[39] Muoio DM, Way JM, Tanner CJ, Winegar DA, Kliewer SA, Houmard JA, et al. Peroxisome proliferator-activated receptor-alpha regulates fatty acid utilization in primary human skeletal muscle cells. Diabetes. 2002;51:901–9. 10.2337/diabetes.51.4.901. 11916905 10.2337/diabetes.51.4.901

[40] Neels JG, Grimaldi PA. Physiological functions of peroxisome proliferator-activated receptor beta. Physiol Rev. 2014;94:795–858. 10.1152/physrev.00027.2013. 24987006 10.1152/physrev.00027.2013

[41] Ma X, Xiao L, Liu L, Ye L, Su P, Bi E, et al. CD36-mediated ferroptosis dampens intratumoral CD8(+) T-cell effector function and impairs their antitumor ability. Cell Metab. 2021;33:1001–12. 10.1016/j.cmet.2021.02.015. e1005 33691090 10.1016/j.cmet.2021.02.015 PMC8102368

[42] Xu S, Chaudhary O, Rodríguez-Morales P, Sun X, Chen D, Zappasodi R, et al. Uptake of oxidized lipids by the scavenger receptor CD36 promotes lipid peroxidation and dysfunction in CD8(+) T cells in tumors. Immunity. 2021;54:1561–77. 10.1016/j.immuni.2021.05.003. e1567 34102100 10.1016/j.immuni.2021.05.003 PMC9273026

[43] MacIver NJ, Michalek RD, Rathmell JC. Metabolic regulation of T lymphocytes. Annu Rev Immunol. 2013;31:259–83. 10.1146/annurev-immunol-032712-095956. 23298210 10.1146/annurev-immunol-032712-095956 PMC3606674

[44] Nicoli F, Papagno L, Frere JJ, Cabral-Piccin MP, Clave E, Gostick E, et al. Naïve CD8(+) T Cells Engage a Versatile Metabolic Program Upon Activation in Humans and Differ Energetically From Memory CD8(+) T Cells. Front Immunol. 2018;9:2736. 10.3389/fimmu.2018.02736. 30619240 10.3389/fimmu.2018.02736 PMC6308131

[45] Frauwirth KA, Riley JL, Harris MH, Parry RV, Rathmell JC, Plas DR, et al. The CD28 signaling pathway regulates glucose metabolism. Immunity. 2002;16:769–77. 10.1016/s1074-7613(02)00323-0. 12121659 10.1016/s1074-7613(02)00323-0

[46] O'sullivan D, van der Windt GJ, Huang SC, Curtis JD, Chang CH, Buck MD, et al. Memory CD8(+) T cells use cell-intrinsic lipolysis to support the metabolic programming necessary for development. Immunity. 2014;41:75–88. 10.1016/j.immuni.2014.06.005. 25001241 10.1016/j.immuni.2014.06.005 PMC4120664

[47] Zhang Y, Kurupati R, Liu L, Zhou XY, Zhang G, Hudaihed A, et al. Enhancing CD8(+) T-Cell Fatty Acid Catabolism within a Metabolically Challenging Tumor Microenvironment Increases the Efficacy of Melanoma Immunotherapy. Cancer Cell. 2017;32:377–91. 10.1016/j.ccell.2017.08.004. 28898698 10.1016/j.ccell.2017.08.004 PMC5751418

[48] Chamoto K, Chowdhury PS, Kumar A, Sonomura K, Matsuda F, Fagarasan S, et al. Mitochondrial activation chemicals synergize with surface receptor PD-1 blockade for T-cell-dependent antitumor activity. Proc Natl Acad Sci USA. 2017;114:E761–E770. 10.1073/pnas.1620433114. 28096382 10.1073/pnas.1620433114 PMC5293087

[49] Scharping NE, Menk AV, Moreci RS, Whetstone RD, Dadey RE, Watkins SC, et al. The Tumor Microenvironment Represses T-Cell Mitochondrial Biogenesis to Drive Intratumoral T-Cell Metabolic Insufficiency and Dysfunction. Immunity. 2016;45:701–3. 10.1016/j.immuni.2016.08.009. 27653602 10.1016/j.immuni.2016.08.009

[50] Hunt EG, Hurst KE, Riesenberg BP, Kennedy AS, Gandy EJ, Andrews AM, et al. Acetyl-CoA carboxylase obstructs CD8(+) T-cell lipid utilization in the tumor microenvironment. Cell Metab. 2024;36:969–83. 10.1016/j.cmet.2024.02.009. 38490211 10.1016/j.cmet.2024.02.009 PMC12010431

[51] Beltra JC, Manne S, Abdel-Hakeem MS, Kurachi M, Giles JR, Chen Z, et al. Developmental Relationships of Four Exhausted CD8(+) T-Cell Subsets Reveals Underlying Transcriptional and Epigenetic Landscape Control Mechanisms. Immunity. 2020;52:825–41. 10.1016/j.immuni.2020.04.014. 32396847 10.1016/j.immuni.2020.04.014 PMC8360766

[52] Im SJ, Hashimoto M, Gerner MY, Lee J, Kissick HT, Burger MC, et al. Defining CD8+ T cells that provide the proliferative burst after PD-1 therapy. Nature. 2016;537:417–21. 10.1038/nature19330. 27501248 10.1038/nature19330 PMC5297183

[53] Gabriel SS, Tsui C, Chisanga D, Weber F, Llano-León M, Gubser PM, et al. Transforming growth factor-beta-regulated mTOR activity preserves cellular metabolism to maintain long-term T-cell responses in chronic infection. Immunity. 2021;54:1698–714. 10.1016/j.immuni.2021.06.007. 34233154 10.1016/j.immuni.2021.06.007

[54] Silverstein RL, Febbraio M. CD36, a scavenger receptor involved in immunity, metabolism, angiogenesis, and behavior. Sci Signal. 2009;2:re3 10.1126/scisignal.272re3. 19471024 10.1126/scisignal.272re3 PMC2811062

[55] Storch J, Corsico B. The emerging functions and mechanisms of mammalian fatty acid-binding proteins. Annu Rev Nutr. 2008;28:73–95. 10.1146/annurev.nutr.27.061406.093710. 18435590 10.1146/annurev.nutr.27.061406.093710

[56] Liu F, Liu W, Zhou S, Yang C, Tian M, Jia G, et al. Identification of FABP5 as an immunometabolic marker in human hepatocellular carcinoma. J Immunother Cancer. 2020;8:e000501. 10.1136/jitc-2019-000501. 32611686 10.1136/jitc-2019-000501 PMC7332195

[57] Frizzell H, Fonseca R, Christo SN, Evrard M, Cruz-Gomez S, Zanluqui NG, et al. Organ-specific isoform selection of fatty acid-binding proteins in tissue-resident lymphocytes. Sci Immunol. 2020;5, 10.1126/sciimmunol.aay9283. 10.1126/sciimmunol.aay9283 32245887

[58] Varanasi SK, Kumar SV, Rouse BT. Determinants of Tissue-Specific Metabolic Adaptation of T Cells. Cell Metab. 2020;32:908–19. 10.1016/j.cmet.2020.10.013. 33181092 10.1016/j.cmet.2020.10.013 PMC7710599

[59] Pan Y, Tian T, Park CO, Lofftus SY, Mei S, Liu X, et al. Survival of tissue-resident memory T cells requires exogenous lipid uptake and metabolism. Nature. 2017;543:252–6. 10.1038/nature21379. 28219080 10.1038/nature21379 PMC5509051

[60] Stahl A, Gimeno RE, Tartaglia LA, Lodish HF. Fatty acid transport proteins: a current view of a growing family. Trends Endocrinol Metab. 2001;12:266–73. 10.1016/s1043-2760(01)00427-1. 11445444 10.1016/s1043-2760(01)00427-1

[61] Gudgeon N, Giles H, Bishop EL, Fulton-Ward T, Escribano-Gonzalez C, Munford H, et al. Uptake of long-chain fatty acids from the bone marrow suppresses CD8+ T-cell metabolism and function in multiple myeloma. Blood Adv. 2023;7:6035–47. 10.1182/bloodadvances.2023009890. 37276076 10.1182/bloodadvances.2023009890 PMC10582277

[62] Bonacina F, Moregola A, Svecla M, Coe D, Uboldi P, Fraire S, et al. The low-density lipoprotein receptor-mTORC1 axis coordinates CD8+ T-cell activation. J Cell Biol. 2022;221, 10.1083/jcb.202202011. 10.1083/jcb.202202011 PMC9499829 36129440

[63] Yuan J, Cai T, Zheng X, Ren Y, Qi J, Lu X, et al. Potentiating CD8(+) T-cell antitumor activity by inhibiting PCSK9 to promote LDLR-mediated TCR recycling and signaling. Protein Cell. 2021;12:240–60. 10.1007/s13238-021-00821-2. 33606190 10.1007/s13238-021-00821-2 PMC8018994

[64] Lim SA, Su W, Chapman NM, Chi H. Lipid metabolism in T-cell signaling and function. Nat Chem Biol. 2022;18:470–81. 10.1038/s41589-022-01017-3. 35484263 10.1038/s41589-022-01017-3 PMC11103273

[65] Chowdhury S, Kar A, Bhowmik D, Gautam A, Basak D, Sarkar I, et al. Intracellular Acetyl-CoA Potentiates the Therapeutic Efficacy of Antitumor CD8+ T Cells. Cancer Res. 2022;82:2640–55. 10.1158/0008-5472.CAN-21-4052. 35648389 10.1158/0008-5472.CAN-21-4052 PMC7613107

[66] Shimano H, Sato R. SREBP-regulated lipid metabolism: convergent physiology - divergent pathophysiology. Nat Rev Endocrinol. 2017;13:710–30. 10.1038/nrendo.2017.91. 28849786 10.1038/nrendo.2017.91

[67] Liu C, Chikina M, Deshpande R, Menk AV, Wang T, Tabib T, et al. Treg Cells Promote the SREBP1-Dependent Metabolic Fitness of Tumor-Promoting Macrophages via Repression of CD8(+) T-Cell-Derived Interferon-gamma. Immunity. 2019;51:381–97. 10.1016/j.immuni.2019.06.017. 31350177 10.1016/j.immuni.2019.06.017 PMC6703933

[68] Reinfeld BI, Madden MZ, Wolf MM, Chytil A, Bader JE, Patterson AR, et al. Cell-programmed nutrient partitioning in the tumor microenvironment. Nature. 2021;593:282–8. 10.1038/s41586-021-03442-1. 33828302 10.1038/s41586-021-03442-1 PMC8122068

[69] Zhang S, Lv K, Liu Z, Zhao R, Li F. Fatty acid metabolism of immune cells: a new target of tumor immunotherapy. Cell Death Discov. 2024;10:39. 10.1038/s41420-024-01807-9. 38245525 10.1038/s41420-024-01807-9 PMC10799907

[70] Evans RM, Barish GD, Wang YX. PPARs and the complex journey to obesity. Nat Med. 2004;10:355–61. 10.1038/nm1025. 15057233 10.1038/nm1025

[71] Chowdhury PS, Chamoto K, Kumar A, Honjo T. PPAR-Induced Fatty Acid Oxidation in T Cells Increases the Number of Tumor-Reactive CD8(+) T Cells and Facilitates Anti-PD-1 Therapy. Cancer Immunol Res. 2018;6:1375–87. 10.1158/2326-6066.CIR-18-0095. 30143538 10.1158/2326-6066.CIR-18-0095

[72] Yu H, Pardoll D, Jove R. STATs in cancer inflammation and immunity: a leading role for STAT3. Nat Rev Cancer. 2009;9:798–809. 10.1038/nrc2734. 19851315 10.1038/nrc2734 PMC4856025

[73] Sun Q, Zhao X, Li R, Liu D, Pan B, Xie B, et al. STAT3 regulates CD8+ T-cell differentiation and functions in cancer and acute infection. J Exp Med. 2023;220, 10.1084/jem.20220686. 10.1084/jem.20220686 PMC9884582 36688918

[74] Zhang C, Yue C, Herrmann A, Song J, Egelston C, Wang T, et al. STAT3 Activation-Induced Fatty Acid Oxidation in CD8(+) T Effector Cells Is Critical for Obesity-Promoted Breast Tumor Growth. Cell Metab. 2020;31:148–61. 10.1016/j.cmet.2019.10.013. 31761565 10.1016/j.cmet.2019.10.013 PMC6949402

[75] Xiao L, Ma X, Ye L, Su P, Xiong W, Bi E, et al. IL-9/STAT3/fatty acid oxidation-mediated lipid peroxidation contributes to Tc9 cell longevity and enhanced antitumor activity. J Clin Invest. 2022;132, 10.1172/JCI153247. 10.1172/JCI153247 PMC8970676 35192544

[76] Schlaepfer IR, Joshi M. CPT1A-mediated Fat Oxidation, Mechanisms, and Therapeutic Potential. Endocrinology. 2020;161, 10.1210/endocr/bqz046. 10.1210/endocr/bqz046 31900483

[77] Raud B, Roy DG, Divakaruni AS, Tarasenko TN, Franke R, Ma EH, et al. Etomoxir Actions on Regulatory and Memory T Cells Are Independent of Cpt1a-Mediated Fatty Acid Oxidation. Cell Metab. 2018;28:504–15. 10.1016/j.cmet.2018.06.002. 30043753 10.1016/j.cmet.2018.06.002 PMC6747686

[78] Coutzac C, Jouniaux JM, Paci A, Schmidt J, Mallardo D, Seck A, et al. Systemic short-chain fatty acids limit antitumor effect of CTLA-4 blockade in hosts with cancer. Nat Commun. 2020;11:2168. 10.1038/s41467-020-16079-x. 32358520 10.1038/s41467-020-16079-x PMC7195489

[79] Luu M, Riester Z, Baldrich A, Reichardt N, Yuille S, Busetti A, et al. Microbial short-chain fatty acids modulate CD8(+) T-cell responses and improve adoptive immunotherapy for cancer. Nat Commun. 2021;12:4077. 10.1038/s41467-021-24331-1. 34210970 10.1038/s41467-021-24331-1 PMC8249424

[80] He Y, Fu L, Li Y, Wang W, Gong M, Zhang J, et al. Gut microbial metabolites facilitate anticancer therapy efficacy by modulating cytotoxic CD8(+) T-cell immunity. Cell Metab. 2021;33:988–1000. 10.1016/j.cmet.2021.03.002. 33761313 10.1016/j.cmet.2021.03.002

[81] Bachem A, Makhlouf C, Binger KJ, de Souza DP, Tull D, Hochheiser K, et al. Microbiota-Derived Short-Chain Fatty Acids Promote the Memory Potential of Antigen-Activated CD8(+) T Cells. Immunity. 2019;51:285–97. 10.1016/j.immuni.2019.06.002. 31272808 10.1016/j.immuni.2019.06.002

[82] Manzo T, Prentice BM, Anderson KG, Raman A, Schalck A, Codreanu GS, et al. Accumulation of long-chain fatty acids in the tumor microenvironment drives dysfunction in intrapancreatic CD8+ T cells. J Exp Med. 2020;217, 10.1084/jem.20191920. 10.1084/jem.20191920 PMC7398173 32491160

[83] Lin Y, Li X, Shan H, Gao J, Yang Y, Jiang L, et al. Scd-1 deficiency promotes the differentiation of CD8(+) T effector. Front Cell Infect Microbiol. 2024;14:1325390. 10.3389/fcimb.2024.1325390. 38379772 10.3389/fcimb.2024.1325390 PMC10876803

[84] Jin R, Hao J, Yi Y, Yin D, Hua Y, Li X, et al. Dietary Fats High in Linoleic Acids Impair Antitumor T-cell Responses by Inducing E-FABP-Mediated Mitochondrial Dysfunction. Cancer Res. 2021;81:5296–310. 10.1158/0008-5472.CAN-21-0757. 34400394 10.1158/0008-5472.CAN-21-0757 PMC8530923

[85] Nava Lauson CB, Tiberti S, Corsetto PA, Conte F, Tyagi P, Machwirth M, et al. Linoleic acid potentiates CD8(+) T-cell metabolic fitness and antitumor immunity. Cell Metab. 2023;35:633–50. 10.1016/j.cmet.2023.02.013. 36898381 10.1016/j.cmet.2023.02.013

[86] Ye Z, Shen Y, Jin K, Qiu J, Hu B, Jadhav RR, et al. Arachidonic acid-regulated calcium signaling in T cells from patients with rheumatoid arthritis promotes synovial inflammation. Nat Commun. 2021;12:907. 10.1038/s41467-021-21242-z. 33568645 10.1038/s41467-021-21242-z PMC7875984

[87] Liao P, Wang W, Wang W, Kryczek I, Li X, Bian Y, et al. CD8(+) T cells and fatty acids orchestrate tumor ferroptosis and immunity via ACSL4. Cancer Cell. 2022;40:365–78. 10.1016/j.ccell.2022.02.003. 35216678 10.1016/j.ccell.2022.02.003 PMC9007863

[88] Feng M, Liu X, Hao X, Ren Y, Dong G, Tian J, et al. Fatty Acids Support the Fitness and Functionality of Tumor-Resident CD8+ T Cells by Maintaining SCML4 Expression. Cancer Res. 2023;83:3368–84. 10.1158/0008-5472.CAN-23-0287. 37610617 10.1158/0008-5472.CAN-23-0287

[89] Morotti M, Grimm AJ, Hope HC, Arnaud M, Desbuisson M, Rayroux N, et al. PGE(2) inhibits TIL expansion by disrupting IL-2 signaling and mitochondrial function. Nature. 2024;629:426–34. 10.1038/s41586-024-07352-w. 38658764 10.1038/s41586-024-07352-w PMC11078736

[90] Lacher SB, Dörr J, de Almeida GP, Hönninger J, Bayerl F, Hirschberger A, et al. PGE(2) limits effector expansion of tumor-infiltrating stem-like CD8(+) T cells. Nature. 2024;629:417–25. 10.1038/s41586-024-07254-x. 38658748 10.1038/s41586-024-07254-x PMC11078747

[91] Chapkin RS, Davidson LA, Ly L, Weeks BR, Lupton JR, McMurray DN. Immunomodulatory effects of (n-3) fatty acids: putative link to inflammation and colon cancer. J Nutr. 2007;137:200S–204S. 10.1093/jn/137.1.200S. 17182826 10.1093/jn/137.1.200S

[92] Liu L, Jin R, Hao J, Zeng J, Yin D, Yi Y, et al. Consumption of the Fish Oil High-Fat Diet Uncouples Obesity and Mammary Tumor Growth through Induction of Reactive Oxygen Species in Protumor Macrophages. Cancer Res. 2020;80:2564–74. 10.1158/0008-5472.CAN-19-3184. 32213543 10.1158/0008-5472.CAN-19-3184 PMC7299802

[93] Bietz A, Zhu H, Xue M, Xu C. Cholesterol Metabolism in T Cells. Front Immunol. 2017;8:1664. 10.3389/fimmu.2017.01664. 29230226 10.3389/fimmu.2017.01664 PMC5711771

[94] Liu X, Bao X, Hu M, Chang H, Jiao M, Cheng J, et al. Inhibition of PCSK9 potentiates immune checkpoint therapy for cancer. Nature. 2020;588:693–8. 10.1038/s41586-020-2911-7. 33177715 10.1038/s41586-020-2911-7 PMC7770056

[95] Bian X, Liu R, Meng Y, Xing D, Xu D, Lu Z. Lipid metabolism and cancer. J Exp Med. 2021;218, 10.1084/jem.20201606. 10.1084/jem.20201606 PMC7754673 33601415

[96] Yang W, Bai Y, Xiong Y, Zhang J, Chen S, Zheng X, et al. Potentiating the antitumour response of CD8(+) T cells by modulating cholesterol metabolism. Nature. 2016;531:651–5. 10.1038/nature17412. 26982734 10.1038/nature17412 PMC4851431

[97] Ma X, Bi E, Huang C, Lu Y, Xue G, Guo X, et al. Cholesterol negatively regulates IL-9-producing CD8(+) T-cell differentiation and antitumor activity. J Exp Med. 2018;215:1555–69. 10.1084/jem.20171576. 29743292 10.1084/jem.20171576 PMC5987919

[98] Baek AE, Yu YA, He S, Wardell SE, Chang CY, Kwon S, et al. The cholesterol metabolite 27 hydroxycholesterol facilitates breast cancer metastasis through its actions on immune cells. Nat Commun. 2017;8:864. 10.1038/s41467-017-00910-z. 29021522 10.1038/s41467-017-00910-z PMC5636879

[99] Su P, Wang Q, Bi E, Ma X, Liu L, Yang M, et al. Enhanced Lipid Accumulation and Metabolism Are Required for the Differentiation and Activation of Tumor-Associated Macrophages. Cancer Res. 2020;80:1438–50. 10.1158/0008-5472.CAN-19-2994. 32015091 10.1158/0008-5472.CAN-19-2994 PMC7127942

[100] York AG, Williams KJ, Argus JP, Zhou QD, Brar G, Vergnes L, et al. Limiting Cholesterol Biosynthetic Flux Spontaneously Engages Type I IFN Signaling. Cell. 2015;163:1716–29. 10.1016/j.cell.2015.11.045. 26686653 10.1016/j.cell.2015.11.045 PMC4783382

[101] Ma X, Bi E, Lu Y, Su P, Huang C, Liu L, et al. Cholesterol Induces CD8(+) T-Cell Exhaustion in the Tumor Microenvironment. Cell Metab. 2019;30:143–56. 10.1016/j.cmet.2019.04.002. 31031094 10.1016/j.cmet.2019.04.002 PMC7061417

[102] Yan C, Zheng L, Jiang S, Yang H, Guo J, Jiang LY, et al. Exhaustion-associated cholesterol deficiency dampens the cytotoxic arm of antitumor immunity. Cancer Cell. 2023;41:1276–93. 10.1016/j.ccell.2023.04.016. 37244259 10.1016/j.ccell.2023.04.016

[103] Morad SA, Cabot MC. Ceramide-orchestrated signaling in cancer cells. Nat Rev Cancer. 2013;13:51–65. 10.1038/nrc3398. 23235911 10.1038/nrc3398

[104] Hose M, Günther A, Naser E, Schumacher F, Schönberger T, Falkenstein J, et al. Cell-intrinsic ceramides determine T-cell function during melanoma progression. Elife. 2022;11, 10.7554/eLife.83073. 10.7554/eLife.83073 PMC9699697 36426850

[105] Oda SK, Strauch P, Fujiwara Y, Al-Shami A, Oravecz T, Tigyi G, et al. Lysophosphatidic acid inhibits CD8 T-cell activation and control of tumor progression. Cancer Immunol Res. 2013;1:245–55. 10.1158/2326-6066.CIR-13-0043-T. 24455753 10.1158/2326-6066.CIR-13-0043-T PMC3893823

[106] Turner JA, Fredrickson MA, D'Antonio M, Katsnelson E, MacBeth M, Van Gulick R, et al. Lysophosphatidic acid modulates CD8 T-cell immunosurveillance and metabolism to impair anti-tumor immunity. Nat Commun. 2023;14:3214. 10.1038/s41467-023-38933-4. 37270644 10.1038/s41467-023-38933-4 PMC10239450

[107] Harel M, Ortenberg R, Varanasi SK, Mangalhara KC, Mardamshina M, Markovits E, et al. Proteomics of Melanoma Response to Immunotherapy Reveals Mitochondrial Dependence. Cell. 2019;179:236–50. 10.1016/j.cell.2019.08.012. 31495571 10.1016/j.cell.2019.08.012 PMC7993352

[108] Steinert EM, Vasan K, Chandel NS. Mitochondrial Metabolism Regulation of T-Cell-Mediated Immunity. Annu Rev Immunol. 2021;39:395–416. 10.1146/annurev-immunol-101819-082015. 33902315 10.1146/annurev-immunol-101819-082015 PMC10403253

[109] Dumauthioz N, Tschumi B, Wenes M, Marti B, Wang H, Franco F, et al. Enforced PGC-1alpha expression promotes CD8 T-cell fitness, memory formation and antitumor immunity. Cell Mol Immunol. 2021;18:1761–71. 10.1038/s41423-020-0365-3. 32055005 10.1038/s41423-020-0365-3 PMC8245409

[110] Simula L, Fumagalli M, Vimeux L, Rajnpreht I, Icard P, Birsen G, et al. Mitochondrial metabolism sustains CD8(+) T-cell migration for an efficient infiltration into solid tumors. Nat Commun. 2024;15:2203. 10.1038/s41467-024-46377-7. 38467616 10.1038/s41467-024-46377-7 PMC10928223

[111] Rieusset J. The role of endoplasmic reticulum-mitochondria contact sites in the control of glucose homeostasis: an update. Cell Death Dis. 2018;9:388. 10.1038/s41419-018-0416-1. 29523782 10.1038/s41419-018-0416-1 PMC5844895

[112] Buck MD, O'Sullivan D, Klein Geltink RI, Curtis JD, Chang CH, Sanin DE, et al. Mitochondrial Dynamics Controls T-Cell Fate through Metabolic Programming. Cell. 2016;166:63–76. 10.1016/j.cell.2016.05.035. 27293185 10.1016/j.cell.2016.05.035 PMC4974356

[113] Su LJ, Zhang JH, Gomez H, Murugan R, Hong X, Xu D, et al. Reactive Oxygen Species-Induced Lipid Peroxidation in Apoptosis, Autophagy, and Ferroptosis. Oxid Med Cell Longev. 2019;2019:5080843–13. 10.1155/2019/5080843. 31737171 10.1155/2019/5080843 PMC6815535

[114] Kirtonia A, Sethi G, Garg M. The multifaceted role of reactive oxygen species in tumorigenesis. Cell Mol Life Sci. 2020;77:4459–83. 10.1007/s00018-020-03536-5. 32358622 10.1007/s00018-020-03536-5 PMC11105050

[115] Yarosz EL, Chang CH. The Role of Reactive Oxygen Species in Regulating T-Cell-mediated Immunity and Disease. Immune Netw. 2018;18:e14. 10.4110/in.2018.18.e14. 29503744 10.4110/in.2018.18.e14 PMC5833121

[116] Siska PJ, Beckermann KE, Mason FM, Andrejeva G, Greenplate AR, Sendor AB, et al. Mitochondrial dysregulation and glycolytic insufficiency functionally impair CD8 T cells infiltrating human renal cell carcinoma. JCI Insight. 2017;2, 10.1172/jci.insight.93411. 10.1172/jci.insight.93411 PMC5470888 28614802

[117] Renken S, Nakajima T, Magalhaes I, Mattsson J, Lundqvist A, Arnér ESJ, et al. Targeting of Nrf2 improves antitumoral responses by human NK cells, TIL and CAR T cells during oxidative stress. J Immunother Cancer. 2022;10:e004458. 10.1136/jitc-2021-004458. 35738800 10.1136/jitc-2021-004458 PMC9226989

[118] Flegal KM, Kit BK, Orpana H, Graubard BI. Association of all-cause mortality with overweight and obesity using standard body mass index categories: a systematic review and meta-analysis. JAMA. 2013;309:71–82. 10.1001/jama.2012.113905. 23280227 10.1001/jama.2012.113905 PMC4855514

[119] Deng T, Lyon CJ, Bergin S, Caligiuri MA, Hsueh WA. Obesity, Inflammation, and Cancer. Annu Rev Pathol. 2016;11:421–49. 10.1146/annurev-pathol-012615-044359. 27193454 10.1146/annurev-pathol-012615-044359

[120] Picon-Ruiz M, Morata-Tarifa C, Valle-Goffin JJ, Friedman ER, Slingerland JM. Obesity and adverse breast cancer risk and outcome: Mechanistic insights and strategies for intervention. CA Cancer J Clin. 2017;67:378–97. 10.3322/caac.21405. 28763097 10.3322/caac.21405 PMC5591063

[121] Sun B, Karin M. Obesity, inflammation, and liver cancer. J Hepatol. 2012;56:704–13. 10.1016/j.jhep.2011.09.020. 22120206 10.1016/j.jhep.2011.09.020 PMC3889660

[122] Hale M, Itani F, Buchta CM, Wald G, Bing M, Norian LA. Obesity triggers enhanced MDSC accumulation in murine renal tumors via elevated local production of CCL2. PLoS One. 2015;10:e0118784. 10.1371/journal.pone.0118784. 25769110 10.1371/journal.pone.0118784 PMC4358922

[123] Kolb R, Phan L, Borcherding N, Liu Y, Yuan F, Janowski AM, et al. Obesity-associated NLRC4 inflammasome activation drives breast cancer progression. Nat Commun. 2016;7:13007. 10.1038/ncomms13007. 27708283 10.1038/ncomms13007 PMC5059727

[124] Pingili AK, Chaib M, Sipe LM, Miller EJ, Teng B, Sharma R, et al. Immune checkpoint blockade reprograms systemic immune landscape and tumor microenvironment in obesity-associated breast cancer. Cell Rep. 2021;35:109285. 10.1016/j.celrep.2021.109285. 34161764 10.1016/j.celrep.2021.109285 PMC8574993

[125] Incio J, Liu H, Suboj P, Chin SM, Chen IX, Pinter M, et al. Obesity-Induced Inflammation and Desmoplasia Promote Pancreatic Cancer Progression and Resistance to Chemotherapy. Cancer Discov. 2016;6:852–69. 10.1158/2159-8290.CD-15-1177. 27246539 10.1158/2159-8290.CD-15-1177 PMC4972679

[126] McDowell S, Milette S, Doré S, Yu MW, Sorin M, Wilson L, et al. Obesity alters monocyte developmental trajectories to enhance metastasis. J Exp Med. 2023;220, 10.1084/jem.20220509. 10.1084/jem.20220509 PMC10182775 37166450

[127] James BR, Tomanek-Chalkley A, Askeland EJ, Kucaba T, Griffith TS, Norian LA. Diet-induced obesity alters dendritic cell function in the presence and absence of tumor growth. J Immunol. 2012;189:1311–21. 10.4049/jimmunol.1100587. 22745381 10.4049/jimmunol.1100587 PMC3401274

[128] Michelet X, Dyck L, Hogan A, Loftus RM, Duquette D, Wei K, et al. Metabolic reprogramming of natural killer cells in obesity limits antitumor responses. Nat Immunol. 2018;19:1330–40. 10.1038/s41590-018-0251-7. 30420624 10.1038/s41590-018-0251-7

[129] Ringel AE, Drijvers JM, Baker GJ, Catozzi A, García-Cañaveras JC, Gassaway BM, et al. Obesity Shapes Metabolism in the Tumor Microenvironment to Suppress Anti-Tumor Immunity. Cell. 2020;183:1848–66. 10.1016/j.cell.2020.11.009 33301708 10.1016/j.cell.2020.11.009 PMC8064125

[130] Dyck L et al. Suppressive effects of the obese tumor microenvironment on CD8 T-cell infiltration and effector function. J Exp Med. 2022;219, 10.1084/jem.20210042. 10.1084/jem.20210042 PMC8932531 35103755

[131] Porsche CE, Delproposto JB, Geletka L, O’Rourke R, Lumeng CN. Obesity results in adipose tissue T-cell exhaustion. JCI Insight. 2021;6, 10.1172/jci.insight.139793. 10.1172/jci.insight.139793 PMC8119198 33724954

[132] Liddle DM, Monk JM, Hutchinson AL, Ma DWL, Robinson LE. CD8(+) T-cell/adipocyte inflammatory cross talk and ensuing M1 macrophage polarization are reduced by fish-oil-derived n-3 polyunsaturated fatty acids, in part by a TNF-alpha-dependent mechanism. J Nutr Biochem. 2020;76:108243. 10.1016/j.jnutbio.2019.108243. 31760229 10.1016/j.jnutbio.2019.108243

[133] Kang KW, Kim S, Cho YB, Ryu SR, Seo YJ, Lee SM. Endogenous n-3 Polyunsaturated Fatty Acids Are Beneficial to Dampen CD8(+) T-Cell-Mediated Inflammatory Response upon the Viral Infection in Mice. Int J Mol Sci. 2019;20:4510. 10.3390/ijms20184510. 31547227 10.3390/ijms20184510 PMC6770599

[134] Wang JY, Wang QW, Yang XY, Yang W, Li DR, Jin JY, et al. GLP-1 receptor agonists for the treatment of obesity: Role as a promising approach. Front Endocrinol. 2023;14:1085799. 10.3389/fendo.2023.1085799. 10.3389/fendo.2023.1085799 PMC9945324 36843578

[135] Piening A, Ebert E, Gottlieb C, Khojandi N, Kuehm LM, Hoft SG, et al. Obesity-related T-cell dysfunction impairs immunosurveillance and increases cancer risk. Nat Commun. 2024;15:2835. 10.1038/s41467-024-47359-5. 38565540 10.1038/s41467-024-47359-5 PMC10987624

[136] Ben Nasr M, Usuelli V, Dellepiane S, Seelam AJ, Fiorentino TV, D'Addio F, et al. Glucagon-like peptide 1 receptor is a T-cell-negative costimulatory molecule. Cell Metab. 2024;36:1302–19. 10.1016/j.cmet.2024.05.001. 38838642 10.1016/j.cmet.2024.05.001

[137] Kado T, Nawaz A, Takikawa A, Usui I, Tobe K. Linkage of CD8(+) T-cell exhaustion with high-fat diet-induced tumorigenesis. Sci Rep. 2019;9:12284. 10.1038/s41598-019-48678-0. 31439906 10.1038/s41598-019-48678-0 PMC6706391

[138] Wang Z, Aguilar EG, Luna JI, Dunai C, Khuat LT, Le CT, et al. Paradoxical effects of obesity on T-cell function during tumor progression and PD-1 checkpoint blockade. Nat Med. 2019;25:141–51. 10.1038/s41591-018-0221-5. 30420753 10.1038/s41591-018-0221-5 PMC6324991

[139] Niu J, Jiang L, Guo W, Shao L, Liu Y, Wang L. The Association between Leptin Level and Breast Cancer: A Meta-Analysis. PLoS One. 2013;8:e67349. 10.1371/journal.pone.0067349. 23826274 10.1371/journal.pone.0067349 PMC3694967

[140] Bader JE, Wolf MM, Lupica-Tondo GL, Madden MZ, Reinfeld BI, Arner EN, et al. Obesity induces PD-1 on macrophages to suppress antitumor immunity. Nature. 2024;630:968–75. 10.1038/s41586-024-07529-3. 38867043 10.1038/s41586-024-07529-3 PMC11456854

[141] Mutlu AS, Duffy J, Wang MC. Lipid metabolism and lipid signals in aging and longevity. Dev Cell. 2021;56:1394–407. 10.1016/j.devcel.2021.03.034. 33891896 10.1016/j.devcel.2021.03.034 PMC8173711

[142] Amorim JA, Coppotelli G, Rolo AP, Palmeira CM, Ross JM, Sinclair DA. Mitochondrial and metabolic dysfunction in aging and age-related diseases. Nat Rev Endocrinol. 2022;18:243–58. 10.1038/s41574-021-00626-7. 35145250 10.1038/s41574-021-00626-7 PMC9059418

[143] Ron-Harel N, Notarangelo G, Ghergurovich JM, Paulo JA, Sage PT, Santos D, et al. Defective respiration and one-carbon metabolism contribute to impaired naïve T-cell activation in aged mice. Proc Natl Acad Sci USA. 2018;115:13347–52. 10.1073/pnas.1804149115. 30530686 10.1073/pnas.1804149115 PMC6310842

[144] Kugel CH, Douglass SM, Webster MR, Kaur A, Liu Q, Yin X, et al. Age Correlates with Response to Anti-PD1, Reflecting Age-Related Differences in Intratumoral Effector and Regulatory T-Cell Populations. Clin Cancer Res. 2018;24:5347–56. 10.1158/1078-0432.CCR-18-1116. 29898988 10.1158/1078-0432.CCR-18-1116 PMC6324578

[145] McHugh D, Gil J. Senescence and aging: Causes, consequences, and therapeutic avenues. J Cell Biol. 2018;217:65–77. 10.1083/jcb.201708092. 29114066 10.1083/jcb.201708092 PMC5748990

[146] Liu, X, Hartman CL, Li L, Albert CJ, Si F, Gao A, et al. Reprogramming lipid metabolism prevents effector T-cell senescence and enhances tumor immunotherapy. Sci Transl Med. 2021;13, 10.1126/scitranslmed.aaz6314. 10.1126/scitranslmed.aaz6314 PMC12040281 33790024

[147] Argiles JM, Lopez-Soriano FJ, Stemmler B, Busquets S. Cancer-associated cachexia - understanding the tumor macroenvironment and microenvironment to improve management. Nat Rev Clin Oncol. 2023;20:250–64. 10.1038/s41571-023-00734-5. 36806788 10.1038/s41571-023-00734-5

[148] Fang R, Yan L, Liao Z. Abnormal lipid metabolism in cancer-associated cachexia and potential therapy strategy. Front Oncol. 2023;13:1123567. 10.3389/fonc.2023.1123567. 37205195 10.3389/fonc.2023.1123567 PMC10185845

[149] Narsale A, Moya R, Ma J, Anderson LJ, Wu D, Garcia JM, et al. Cancer-driven changes link T-cell frequency to muscle strength in people with cancer: a pilot study. J Cachexia Sarcopenia Muscle. 2019;10:827–43. 10.1002/jcsm.12424. 30977974 10.1002/jcsm.12424 PMC6711422

[150] Baazim H, Schweiger M, Moschinger M, Xu H, Scherer T, Popa A, et al. CD8(+) T cells induce cachexia during chronic viral infection. Nat Immunol. 2019;20:701–10. 10.1038/s41590-019-0397-y. 31110314 10.1038/s41590-019-0397-y PMC6531346

[151] Rundqvist, H, Veliça P, Barbieri L, Gameiro PA, Bargiela D, Gojkovic M, et al. Cytotoxic T cells mediate exercise-induced reductions in tumor growth. Elife. 2020;9, 10.7554/eLife.59996. 10.7554/eLife.59996 PMC7584454 33095157

[152] Voltarelli VA, Amano MT, Tobias GC, Borges GS, Oliveira da Paixão A, Pereira MG, et al. Moderate-intensity aerobic exercise training improves CD8(+) tumor-infiltrating lymphocytes effector function by reducing mitochondrial loss. iScience. 2024;27:110121. 10.1016/j.isci.2024.110121. 38957793 10.1016/j.isci.2024.110121 PMC11217614

[153] Gomes-Santos IL, Amoozgar Z, Kumar AS, Ho WW, Roh K, Talele NP, et al. Exercise Training Improves Tumor Control by Increasing CD8(+) T-cell Infiltration via CXCR3 Signaling and Sensitizes Breast Cancer to Immune Checkpoint Blockade. Cancer Immunol Res. 2021;9:765–78. 10.1158/2326-6066.CIR-20-0499. 33839688 10.1158/2326-6066.CIR-20-0499 PMC8295193

[154] Kivimaki M, Bartolomucci A, Kawachi I. The multiple roles of life stress in metabolic disorders. Nat Rev Endocrinol. 2023;19:10–27. 10.1038/s41574-022-00746-8. 36224493 10.1038/s41574-022-00746-8 PMC10817208

[155] Abate M, Citro M, Caputo M, Pisanti S, Martinelli R. Psychological Stress and Cancer: New Evidence of An Increasingly Strong Link. Transl Med UniSa. 2020;23:53–57. 10.37825/2239-9747.1010. 33457324 10.37825/2239-9747.1010 PMC8370516

[156] He XY, Gao Y, Ng D, Michalopoulou E, George S, Adrover JM, et al. Chronic stress increases metastasis via neutrophil-mediated changes to the microenvironment. Cancer Cell. 2024;42:474–86. 10.1016/j.ccell.2024.01.013. 38402610 10.1016/j.ccell.2024.01.013 PMC11300849

[157] Sommershof A, Scheuermann L, Koerner J, Groettrup M. Chronic stress suppresses anti-tumor T(CD8+) responses and tumor regression following cancer immunotherapy in a mouse model of melanoma. Brain Behav Immun. 2017;65:140–9. 10.1016/j.bbi.2017.04.021. 28457810 10.1016/j.bbi.2017.04.021

[158] Globig AM, Zhao S, Roginsky J, Maltez VI, Guiza J, Avina-Ochoa N, et al. The beta(1)-adrenergic receptor links sympathetic nerves to T-cell exhaustion. Nature. 2023;622:383–92. 10.1038/s41586-023-06568-6. 37731001 10.1038/s41586-023-06568-6 PMC10871066

[159] Wang Y, Narasimamurthy R, Qu M, Shi N, Guo H, Xue Y, et al. Circadian regulation of cancer stem cells and the tumor microenvironment during metastasis. Nat Cancer. 2024;5:546–56. 10.1038/s43018-024-00759-4. 38654103 10.1038/s43018-024-00759-4

[160] Peng F, Lu J, Su K, Liu X, Luo H, He B, et al. Oncogenic fatty acid oxidation senses circadian disruption in sleep-deficiency-enhanced tumorigenesis. Cell Metab. 2024;36:1598–618. 10.1016/j.cmet.2024.04.018. 38772364 10.1016/j.cmet.2024.04.018

[161] Wang C, Zeng Q, Gül ZM, Wang S, Pick R, Cheng P, et al. Circadian tumor infiltration and function of CD8(+) T cells dictate immunotherapy efficacy. Cell. 2024;187:2690–702. 10.1016/j.cell.2024.04.015. 38723627 10.1016/j.cell.2024.04.015

[162] Patsoukis N, Bardhan K, Chatterjee P, Sari D, Liu B, Bell LN, et al. PD-1 alters T-cell metabolic reprogramming by inhibiting glycolysis and promoting lipolysis and fatty acid oxidation. Nat Commun. 2015;6:6692 10.1038/ncomms7692. 25809635 10.1038/ncomms7692 PMC4389235

[163] Pascual G, Avgustinova A, Mejetta S, Martín M, Castellanos A, Attolini CS, et al. Targeting metastasis-initiating cells through the fatty acid receptor CD36. Nature. 2017;541:41–45. 10.1038/nature20791. 27974793 10.1038/nature20791

[164] Powell JD, Delgoffe GM. The mammalian target of rapamycin: linking T-cell differentiation, function, and metabolism. Immunity. 2010;33:301–11. 10.1016/j.immuni.2010.09.002. 20870173 10.1016/j.immuni.2010.09.002 PMC2962404

[165] Crompton JG, Sukumar M, Roychoudhuri R, Clever D, Gros A, Eil RL, et al. Akt inhibition enhances expansion of potent tumor-specific lymphocytes with memory cell characteristics. Cancer Res. 2015;75:296–305. 10.1158/0008-5472.CAN-14-2277. 25432172 10.1158/0008-5472.CAN-14-2277 PMC4384335

[166] Saibil SD, St Paul M, Laister RC, Garcia-Batres CR, Israni-Winger K, Elford AR, et al. Activation of Peroxisome Proliferator-Activated Receptors alpha and delta Synergizes with Inflammatory Signals to Enhance Adoptive Cell Therapy. Cancer Res. 2019;79:445–51. 10.1158/0008-5472.CAN-17-3053. 30573521 10.1158/0008-5472.CAN-17-3053

[167] Mao, W, Cai Y, Chen D, Jiang G, Xu Y, Chen R, et al. Statin shapes inflamed tumor microenvironment and enhances immune checkpoint blockade in non-small cell lung cancer. JCI Insight. 2022;7, 10.1172/jci.insight.161940. 10.1172/jci.insight.161940 PMC9675559 35943796

[168] Santoni M, Massari F, Matrana MR, Basso U, De Giorgi U, Aurilio G, et al. Statin use improves the efficacy of nivolumab in patients with advanced renal cell carcinoma. Eur J Cancer. 2022;172:191–8. 10.1016/j.ejca.2022.04.035. 35780525 10.1016/j.ejca.2022.04.035

[169] Kansal V, Burnham AJ, Kinney B, Saba NF, Paulos C, Lesinski GB, et al. Statin drugs enhance responses to immune checkpoint blockade in head and neck cancer models. J Immunother Cancer. 2023;11:e005940. 10.1136/jitc-2022-005940. 36650022 10.1136/jitc-2022-005940 PMC9853267

[170] Xia Y, Xie Y, Yu Z, Xiao H, Jiang G, Zhou X, et al. The Mevalonate Pathway Is a Druggable Target for Vaccine Adjuvant Discovery. Cell. 2018;175:1059–73. 10.1016/j.cell.2018.08.070. 30270039 10.1016/j.cell.2018.08.070

[171] Jiang X, Stockwell BR, Conrad M. Ferroptosis: mechanisms, biology and role in disease. Nat Rev Mol Cell Biol. 2021;22:266–82. 10.1038/s41580-020-00324-8. 33495651 10.1038/s41580-020-00324-8 PMC8142022

[172] Wang W, Green M, Choi JE, Gijón M, Kennedy PD, Johnson JK, et al. CD8(+) T cells regulate tumor ferroptosis during cancer immunotherapy. Nature. 2019;569:270–4. 10.1038/s41586-019-1170-y. 31043744 10.1038/s41586-019-1170-y PMC6533917

[173] Drijvers JM, Gillis JE, Muijlwijk T, Nguyen TH, Gaudiano EF, Harris IS, et al. Pharmacologic Screening Identifies Metabolic Vulnerabilities of CD8(+) T Cells. Cancer Immunol Res. 2021;9:184–99. 10.1158/2326-6066.CIR-20-0384. 33277233 10.1158/2326-6066.CIR-20-0384 PMC7864883

[174] Prendeville H, Lynch L. Diet, lipids, and antitumor immunity. Cell Mol Immunol. 2022;19:432–44. 10.1038/s41423-021-00781-x. 34983949 10.1038/s41423-021-00781-x PMC8891265

[175] Lien EC, Westermark AM, Zhang Y, Yuan C, Li Z, Lau AN, et al. Low glycemic diets alter lipid metabolism to influence tumor growth. Nature. 2021;599:302–7. 10.1038/s41586-021-04049-2. 34671163 10.1038/s41586-021-04049-2 PMC8628459

[176] de Groot S, Lugtenberg RT, Cohen D, Welters M, Ehsan I, Vreeswijk M, et al. Fasting mimicking diet as an adjunct to neoadjuvant chemotherapy for breast cancer in the multicenter randomized phase 2 DIRECT trial. Nat Commun. 2020;11:3083. 10.1038/s41467-020-16138-3. 32576828 10.1038/s41467-020-16138-3 PMC7311547

[177] Wang G, Li J, Bojmar L, Chen H, Li Z, Tobias GC, et al. Tumor extracellular vesicles and particles induce liver metabolic dysfunction. Nature. 2023;618:374–82. 10.1038/s41586-023-06114-4. 37225988 10.1038/s41586-023-06114-4 PMC10330936

[178] Wang Z, Kim SY, Tu W, Kim J, Xu A, Yang YM, et al. Extracellular vesicles in fatty liver promote a metastatic tumor microenvironment. Cell Metab. 2023;35:1209–26. 10.1016/j.cmet.2023.04.013. 37172577 10.1016/j.cmet.2023.04.013 PMC10524732

[179] Klein Geltink RI, Edwards-Hicks J, Apostolova P, O’Sullivan D, Sanin DE, Patterson AE, et al. Metabolic conditioning of CD8(+) effector T cells for adoptive cell therapy. Nat Metab. 2020;2:703–16. 10.1038/s42255-020-0256-z. 32747793 10.1038/s42255-020-0256-z PMC10863625

[180] Li, C, Phoon YP, Karlinsey K, Tian YF, Thapaliya S, Thongkum A, et al. A high OXPHOS CD8 T-cell subset is predictive of immunotherapy resistance in melanoma patients. J Exp Med. 2022;219, 10.1084/jem.20202084. 10.1084/jem.20202084 PMC8611729 34807232

[181] Sun C, Wang A, Zhou Y, Chen P, Wang X, Huang J, et al. Spatially resolved multiomics highlights cell-specific metabolic remodeling and interactions in gastric cancer. Nat Commun. 2023;14:2692. 10.1038/s41467-023-38360-5. 37164975 10.1038/s41467-023-38360-5 PMC10172194

